# An Ethylene‐Response Factor LlERF092 Coordinates With LlETO1 to Improve Thermotolerance by Activating *LlMBF1c* in Lily

**DOI:** 10.1111/pbi.70269

**Published:** 2025-07-22

**Authors:** Jun Xiang, Xue Gong, Qianqian Fang, Liping Ding, Yinyi Zhang, Sujuan Xu, Ting Li, Man He, Ze Wu, Nianjun Teng

**Affiliations:** ^1^ Key Laboratory of Landscaping Agriculture/Key Laboratory of Flower Biology and Germplasm Innovation, Ministry of Agriculture and Rural Affairs College of Horticulture, Nanjing Agricultural University Nanjing China; ^2^ Lily Science and Technology Backyard Qixia of Jiangsu/Jiangsu Graduate Workstation Nanjing China

**Keywords:** ethylene, lily, LlERF092, LlETO1, thermotolerance

## Abstract

Multiprotein bridging factor 1c (MBF1c) has been shown to play a critical role in plant responses to heat stress. Previous studies have implicated MBF1c roles in ethylene‐mediated thermotolerance; however, the upstream regulatory mechanisms linking MBF1c to this process remain unclear. In this study, an ethylene‐response factor (ERF), LlERF092, was identified as a potential regulator of *LlMBF1c* through a yeast one‐hybrid screening assay. Further investigations revealed that LlERF092 directly bound to the promoter of *LlMBF1c* and activated its transcription. *LlERF092* was rapidly induced by heat stress, and its protein localised to the nucleus. Overexpression of *LlERF092* enhanced the thermotolerance of the transgenic lily plants. Furthermore, immunoprecipitation followed by mass spectrometry (IP‐MS) identified LlETO1 (ETHYLENE OVERPRODUCER 1) as an interacting partner of LlERF092. The expression of *LlETO1* was activated in response to transient heat stress, and the LlETO1‐LlERF092 interaction enhanced the transcriptional activity of LlERF092. Co‐overexpression of *LlERF092* and *LlETO1* enhanced thermotolerance more than the overexpression of either gene alone, while co‐silencing of *LlERF092* and *LlETO1* further reduced thermotolerance compared to silencing each gene individually. Additionally, heat stress promoted ethylene production in lily leaves, and exogenous application of ethephon enhanced thermotolerance. Ethephon treatment also elevated the expression of *LlERF092*, *LlETO1*, and *LlMBF1c*, while their expression was repressed by 1‐MCP under heat stress. In summary, these findings demonstrated that the LlERF092/LlETO1‐LlMBF1c transcriptional cascade mediated ethylene‐dependent thermotolerance in lily under heat stress conditions. This study provides new insights into the molecular mechanisms underlying plant heat stress responses and highlights the role of ethylene signalling in thermotolerance.

## 1 Introduction

Plants have developed intricate and diverse mechanisms to cope with heat stress, due to their inability to alter their position in order to move away from harmful high temperatures. Previous studies have identified several key pathways that plants use to manage heat stress, including the heat stress transcription factor (HSF)‐mediated heat shock protein (HSP) response pathway, the calcium‐dependent calmodulin‐mediated pathway, the hormone‐mediated pathway, the reactive oxygen species (ROS)‐mediated pathway, the unfolded protein (UPR)‐mediated pathway, the epigenetic regulatory pathway, and the non‐coding RNA‐mediated pathway (Zhang et al. 2022; Sugio et al. 2009; Kan et al. 2023). Among these pathways, MBF1c (multiprotein bridging factor 1c) has been identified as a critical regulator of thermotolerance involving HSF‐, hormone‐, and RNA‐mediated pathways (Ohama et al. 2017). Knockout of *MBF1c* reduces the thermotolerance of Arabidopsis, whereas overexpression of *AtMBF1c* enhanced thermotolerance (Suzuki et al. 2005). Further investigation has revealed that AtMBF1c is also involved in specific hormonal pathways involving salicylic acid and ethylene (Suzuki et al. 2005, 2008). Transcriptome analysis of *mbf1c* mutant indicated that AtMBF1c regulated a range of heat‐related genes, including *heat stress factor B2A* (*HSFB2A*), *HSFB2B*, and *dehydration‐responsive element‐binding protein 2A* (*DREB2A*) (Suzuki et al. 2011). Tian et al. (2022) found that *TaMBF1c* knockdown and knockout lines decreased the thermotolerance of *Triticum aestivum* , and further analysis found TaMBF1c interacted with TaG3BP under heat stress. Furthermore, gene expression network analysis suggested that TaMBF1c was tightly linked to the translation of heat shock proteins (Tian et al. 2022). According to Liu et al. (2023), grapes overexpressing *MBF1c* exhibited an increased thermotolerance phenotype, while grapes with MBF1c malfunction had a thermosensitive phenotype (Liu et al. 2023). Variation in the HSFA2 sequence across *Vitis vinifera* ‘Jingxiu’ (heat sensitive) and *V. davidii* ‘Tangwei’ (heat tolerant) varieties transcriptional activities resulted in differences in their ability to activate target genes, including *MBF1c*, which in turn affected differences in varietal heat tolerance (Liu et al. 2023). Overexpressed *MBF1c* of Cucurbitaceae (*CsMBF1c*) enhanced the thermotolerance of cucumber, and the mutants showed thermosensitivity. CsMBF1c interacted with CsDREB2 and nuclear factor Y A1 (CsNFYA1) to regulate thermotolerance of cucumber (Yu et al. 2024). While a number of investigations have attempted to determine how MBF1c mediates thermotolerance, the transcriptional regulatory mechanisms underlying ethylene‐mediated MBF1c thermotolerance remain poorly understood.

Ethylene plays a critical role in regulating plant physiological processes and responding to various environmental stressors (Kazan 2015). Pre‐treatment of Arabidopsis seedlings with 100 μM 1‐aminocyclopropane‐1‐carboxylic acid (ACC) enhanced thermotolerance, while the ethylene‐insensitive mutant *etr‐1* exhibited decreased thermotolerance (Larkindale et al. 2005). Exogenous ACC treatment has also been shown to improve thermotolerance in rice ( *Oryza sativa* ) seedlings, with increased expression of HSFs observed in ACC‐treated seedlings (Wu and Yang 2019). Despite this evidence, the transcriptional regulatory mechanisms by which ethylene improves thermotolerance remain largely unexplored.

Ethylene response factors (ERFs) were key transcriptional regulators mediating ethylene responses and played essential roles in plant adaptation to biotic and abiotic stresses (Hinz et al. 2010; Gasch et al. 2016; Mizoi et al. 2012; Jung et al. 2010; Kong et al. 2023). Overexpression of *ERF012* of *Lilium longiflorum* (*LlERF012*) enhances the thermotolerance of transgenic lily and Arabidopsis. LlERF012 enhances the expression of *HSFA1*, *HSFA2*, *HSFA3A*, and *HSFA3B* by binding to their promoters to increase lily thermotolerance. Moreover, LlERF012 interacts with LlHSFA1, thereby enhancing the transcriptional activity and DNA‐binding capability of LlERF012 (Li et al. 2024). ERF95 has been demonstrated to exert a positive influence on the basal thermotolerance of Arabidopsis and to physically interact with ERF97, functioning downstream of ETHYLENE‐INSENSITIVE 3 (EIN3). Plants with constitutive expression of *ERF95* or *ERF97* have higher basal thermotolerance. Conversely, the quadruple mutants *erf95erf96erf97erf98* show less thermotolerance. In addition, ERF95/ERF97 bind directly to *HSFA2*'s promoter, thereby stimulating the mRNA level of *HSFA2* and enhancing thermotolerance (Huang et al. 2021). The ERF response to heat stress has been reported through the HSF pathway, however the response through other pathways remains to be elucidated.

In this study, we demonstrated that the ethylene response factor LlERF092, derived from lily ( *L. longiflorum* ), contributed positively to thermotolerance. LlERF092 bound directly to the promoter of *LlMBF1c*, activating its expression. Additionally, LlETO1 synergistically interacted with LlERF092 to regulate *LlMBF1c* and improved thermotolerance in lily. Importantly, we confirmed that the function of the LlERF092/LlETO1‐LlMBF1c module in thermotolerance establishment was partially dependent on ethylene signalling. These findings provide novel insights into the molecular mechanisms by which ethylene and ERF‐mediated transcriptional regulation contribute to heat stress response in plants.

## 2 Results

### 2.1 The F2 Fragment is the Key Element for the Heat‐Inducible Activity of the *LlMBF1c* Promoter

**FIGURE 1:**
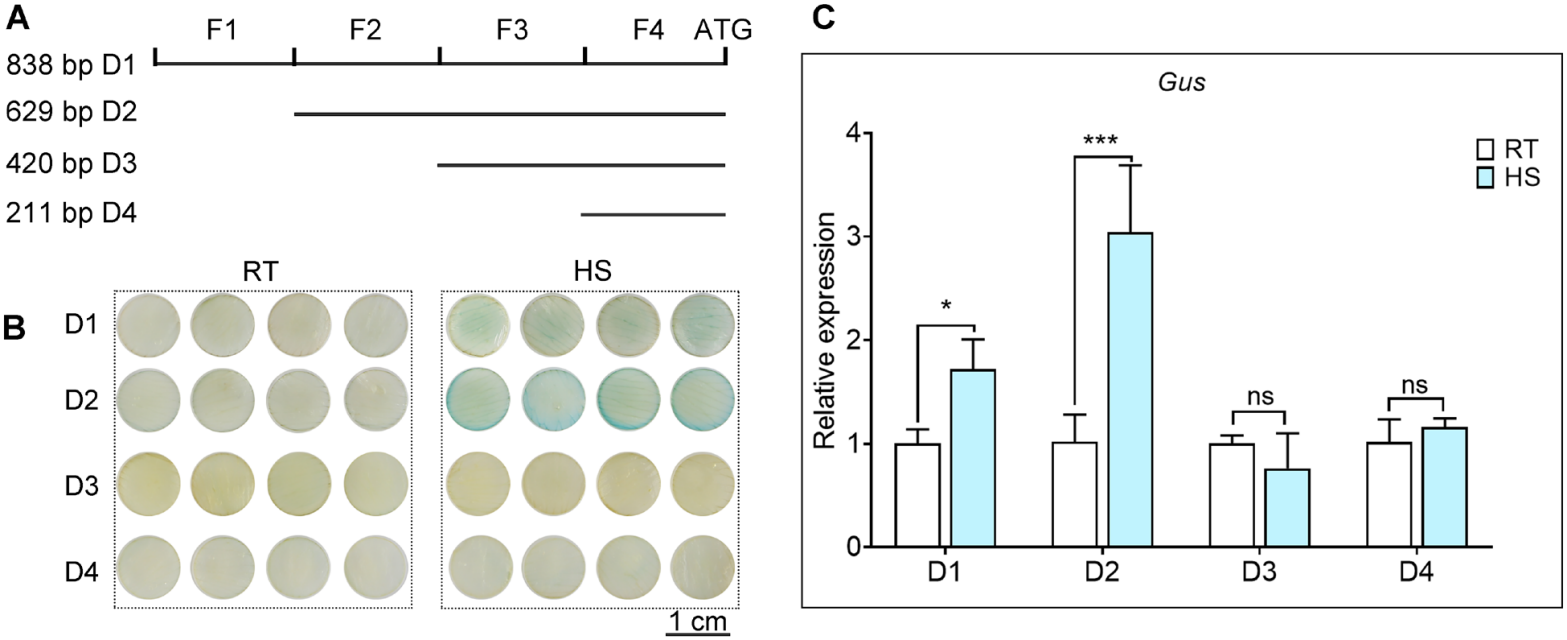
The F2 fragment of the *LlMBF1c* promoter is induced by heat stress. (A) Schematic diagram of the truncation patterns of the *LlMBF1c* promoter. (B) β‐glucuronidase activity analysis of lily petal discs with the different *LlMBF1c* promoter fragments. RT, room temperature, cultured at 22°C; HS, cultured at 37°C for 1 h. The representative image is based on three independent experiments. Scale bar = 1 cm. (C) Quantitative expression analysis of the *GUS* gene in lily petal discs with the different *LlMBF1c* promoter fragments. Data are presented as mean ± SD of three replicates (Student's *t*‐test, *p < 0.05; ***p < 0.001). HS, heat stress at 37°C for 1 h; ns, no significant difference; RT, room temperature (22°C).

Our previous study demonstrated that *LlMBF1c* plays a positive role in the thermotolerance of lily and its promoter activity has been identified as heat‐inducible (Xiang et al. 2024). To determine the heat‐responsive region within the *LlMBF1c* promoter, the promoter was truncated into four fragments (Figure 1A). The β‐glucuronidase (GUS) activity assays revealed that petal discs transformed with the D1 and D2 fragments exhibited enhanced β‐glucuronidase activity under heat stress, whereas petal discs transformed with the D3 and D4 fragments showed no differences in β‐glucuronidase activity between heat‐stressed and untreated conditions (Figure 1B). Further analysis revealed that petal discs containing the F2 fragment (D1 and D2) exhibited β‐glucuronidase activity. Petal discs transformed with D3 and D4, which lost the F2 fragment, showed no difference in β‐glucuronidase activity between RT and heat stress conditions. Similarly, the expression of *GUS* genes in petal discs transformed with D1 and D2 fragments was markedly upregulated upon heat treatment, while those transformed with D3 and D4, which lost the F2 fragment, showed no significant changes in gene expression before or after heat stress (Figure 1C). These results suggested that the F2 fragment of the *LlMBF1c* promoter was specifically induced by heat treatment.

### 2.2 LlERF092 Activates *LlMBF1c* by Binding Its Promoter

**FIGURE 2:**
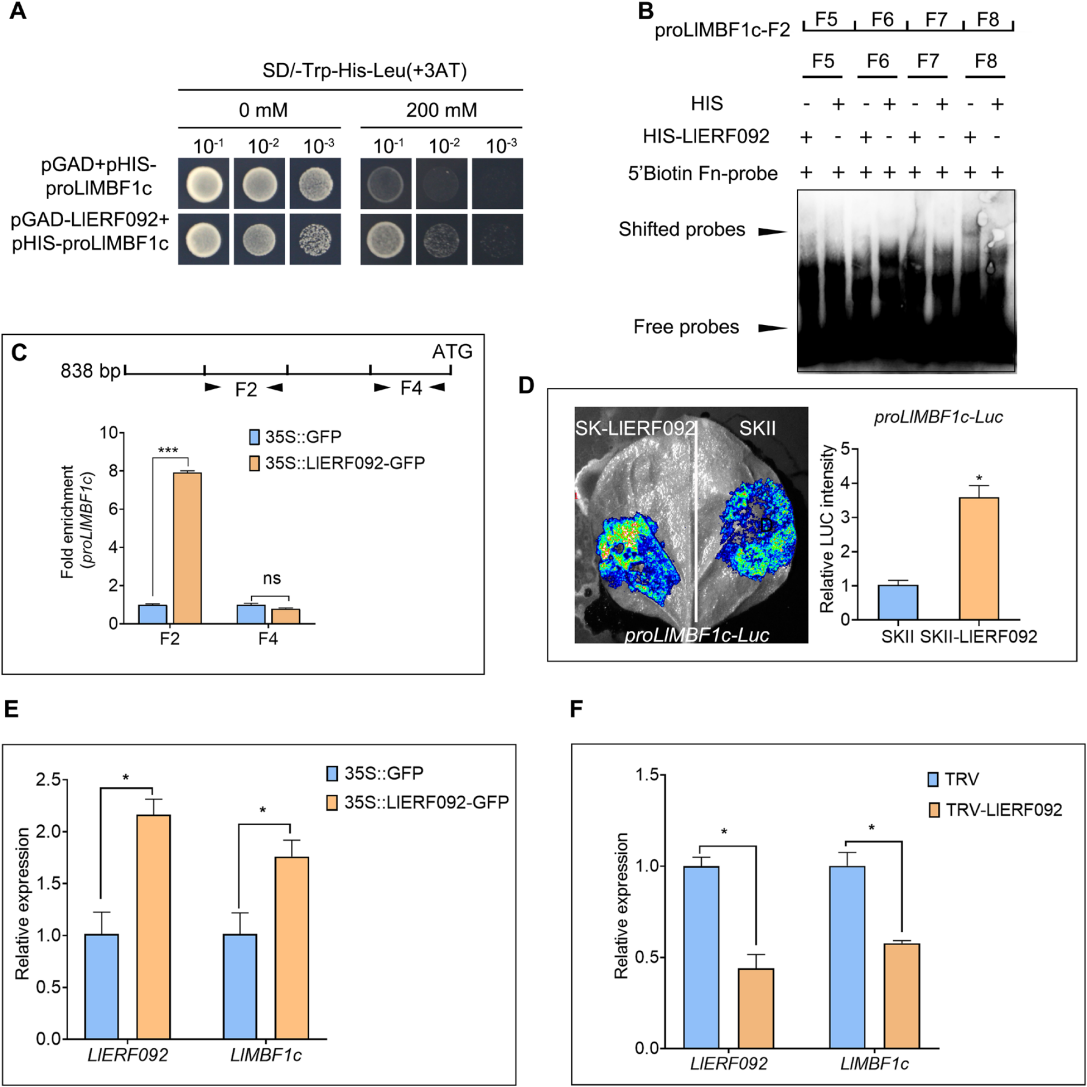
LlERF092 binds to the promoter of *LlMBF1c* and activates its expression. (A) Growth status of transformed yeast cells on SD/‐Trp‐His‐Leu medium with different concentrations of 3‐amino‐1,2,4‐triazole (3‐AT). SD, synthetic dropout medium. (B) An electrophoretic mobility shift analysis of HIS‐LlERF092 and the *LlMBF1c* promoter. Representative image based on three experiments. (C) LlERF092 bound to the F2 fragment of the *LlMBF1c* promoter in vivo. Data are presented as mean ± SD of three replicates (Student's *t*‐test, ***p < 0.001). ns, no significant difference. (D) LlERF092 activated the activity of the *LlMBF1c* promoter in *N. benthamiana*. A representative image derived from three tests; data are presented as mean ± SD (Student's *t*‐test, *p < 0.05). (E) Expression analysis of *LlMBF1c* in *LlERF092* transiently overexpressed lily leaves. 18s rRNA of lily served as the reference gene. Data are shown as the mean ± SD of three replicates (Student's *t*‐test, *p < 0.05). (F) Expression analysis of *LlERF092* and *LlMBF1c* in *LlERF092*‐silenced lily leaves. 18s rRNA of lily served as the reference gene. Data are presented as mean ± SD of three replicates (Student's *t*‐test, *p < 0.05).

To identify the heat‐related regulators of *LlMBF1c*, the yeast one‐hybrid screening assay was performed using pHis2.1‐proLlMBF1cF2 plasmid. The screening for inhibitory concentrations demonstrated that 20 mM 3‐amino‐1,2,4‐triazole (3‐AT) effectively suppressed the self‐activation of the F2 fragment (Figure S1). Using a yeast one‐hybrid screening library targeting the F2 fragment, followed by sequencing analysis, 74 candidate genes were identified (Table S2). We identified an ethylene response factor, ERF092, from these candidate genes. One‐to‐one Y1H assay revealed that LlERF092 was capable of binding to the *LlMBF1c* promoter (Figure 2A) and specifically to the F2 fragment (Figure S2A). In addition, the EMSA assay demonstrated that LlERF092 was able to bind to the probes of the F2 fragment of the *LlMBF1c* promoter (Figure 2B). Further evidence from ChIP‐qPCR experiments demonstrated that LlERF092 bound to the region containing the F2 fragment of the *LlMBF1c* promoter in vivo (Figure 2C). Additionally, transient overexpression of *LlERF092* in *N. benthamiana* enhanced the activity of the *LlMBF1c* promoter (Figure 2D) and its F2 fragment (Figure S2B), as evidenced by a marked increase in relative LUC fluorescence signals in the *LlERF092* overexpression region (Figure 2D; Figure S2C). Furthermore, transient overexpression of *LlERF092* in lily led to an elevation of *LlMBF1c* expression (Figure 2E). Conversely, silencing of *LlERF092* resulted in a reduction in *LlMBF1c* expression (Figure 2F). These findings suggested that LlERF092 was a direct activator of *LlMBF1c*.

### 2.3 Overexpression of *LlERF092* Enhances Thermotolerance in Lily

**FIGURE 3:**
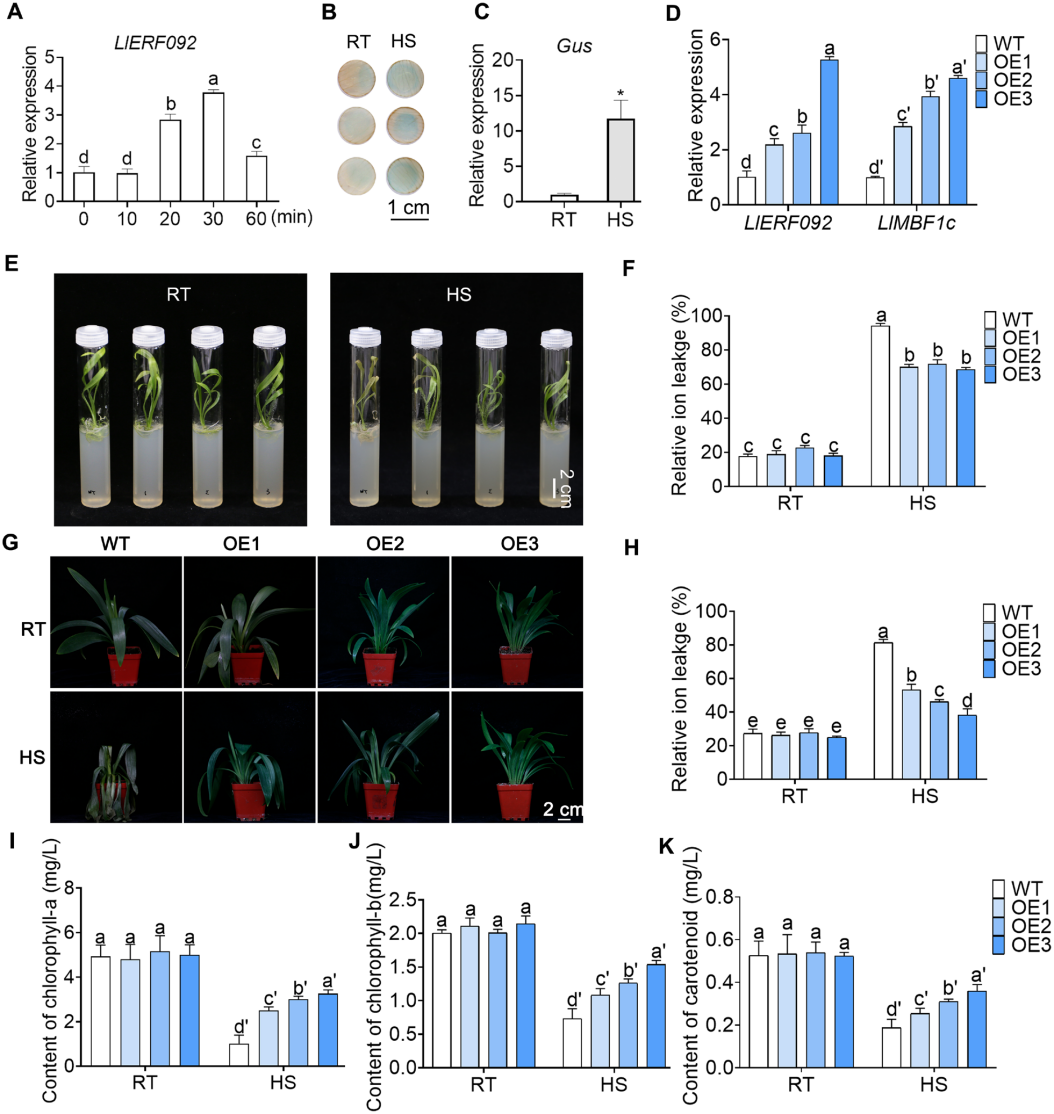
LlERF092 plays a positive role in thermotolerance. (A) Expression analysis of *LlERF092* under heat stress. 18s rRNA of lily served as the reference gene. Data are the mean ± SD of three replicates, with different letters indicating statistically significant difference (Student–Newman–Keuls test, *p* < 0.05). (B) β‐glucuronidase activity analysis in *LlERF092* promoter transformed petal discs. RT, room temperature, cultured at 22°C; HS, cultured at 37°C for 30 min; Scale bar = 1 cm. (C) Expression analysis of the *GUS* gene in *LlERF092* promoter‐transformed petal discs. 18s rRNA of lily served as reference gene. Data are shown as mean ± SD of three replicates (Student's *t*‐test, *p < 0.05). (D) Expression analysis of *LlERF092* and *LlMBF1c* in *LlERF092*‐overexpression lines. The wild type (WT) served as control. 18s rRNA of lily served as the reference gene. Data are presented as mean ± SD of three replicates, with different letters indicating statistically significant difference (Student–Newman–Keuls test, *p* < 0.05). (E) Phenotype analysis of *LlERF092*‐overexpression seedlings under heat stress. Lily bulbs with a diameter of 1 cm were cultured for 3 weeks. RT, room temperature, cultured at 22°C; HS, cultured at 45°C. Scale bar = 2 cm. (F) Relative ion leakage analysis of *LlERF092*‐overexpression seedlings. RT, room temperature, cultured at 22°C; HS, cultured at 45°C. Data are presented as mean ± SD of three replicates, with different letters indicating statistically significant difference (Student–Newman–Keuls test, *p* < 0.05). (G) Phenotype analysis of *LlERF092*‐overexpression lily plants under heat stress. Lily bulbs with a diameter of 1.5 cm were cultured for 5 weeks. RT, room temperature, cultured at 22°C; HS, cultured at 45°C. Scale bar = 2 cm. (H) Relative ion leakage analysis of *LlERF092*‐overexpression lily plants. RT, room temperature, cultured at 22°C; HS, cultured at 45°C. Data are presented as mean ± SD of three replicates, with different letters indicating statistically significant difference (Student–Newman–Keuls test, *p* < 0.05). (I–K) Chlorophyll a (I), chlorophyll b (J), and carotenoid (K) content analysis of transgenic lily plants. RT, room temperature, cultured at 22°C; HS, cultured at 45°C. Data are shown as the mean ± SD of three replicates, with different letters indicating statistically significant difference (Student–Newman–Keuls test, *p* < 0.05).

The ORF of *LlERF092* is 588 bp, encoding a protein of 195 amino acids. Phylogenetic analysis using the protein sequences of the ERF family from Arabidopsis revealed that LlERF092 is closely related to AtERF092, leading to its naming as LlERF092 (Figure S3A). Comparative analyses of the protein sequences of LlERF092 with homologues from *Oryza sativa* (rice), *Capsicum annuum* , *Triticum aestivum* (wheat), *Solanum lycopersicum* (tomato), and Arabidopsis confirmed that LlERF092 contained a typical AP2 domain, identifying it as a member of the AP2/ERF ERF subfamily (Figure S3B). Expression analysis showed that *LlERF092* was rapidly induced at 20 min after heat treatment, peaked at 30 min, and decreased at 60 min (Figure 3A). LlERF092 was localised in the nucleus under 22°C, which remained located in the nucleus under heat stress (Figure S4). LlERF092 had transcriptional activity, and its activation domain was located within 63 amino acids at the N‐terminus and 17 amino acids at the C‐terminus (Figure S5). The β‐galactosidase activity of petal discs transformed with the GUS reporter driven by the *LlERF092* promoter was increased at 30 min after heat treatment, correlating with increased expression of the *GUS* gene (Figure 3B,C). These results suggested that *LlERF092* was quickly induced by heat stress treatment. Transgenic lily lines overexpressing *LlERF092* were generated to further explore the function of LlERF092. The results of the DNA insertion and protein accumulation analysis demonstrated that *LlERF092* had been transferred into the plants (Figure S6). Three overexpression lines (LlERF092‐OE1, LlERF092‐OE2, and LlERF092‐OE3) with elevated transcript levels of *LlERF092* were obtained (Figure 3D). The tissue‐cultured seedlings were used for the thermotolerance test. The results showed that wild‐type (WT) plants were more heat‐sensitive compared to the LlERF092‐OE seedlings (Figure 3E). This thermotolerance increase was accompanied by a lower relative ion leakage (RIL) of the LlERF092‐OE seedlings after heat stress, indicating that *LlERF092* overexpression improved cell membrane stability under heat stress (Figure 3F). Additionally, *LlMBF1c* expression levels were higher in the LlERF092‐OE lines than in WT plants (Figure 3D). ChIP‐qPCR analysis in LlERF092‐OE2 plants also showed that LlERF092 bound to the promoter of *LlMBF1c* in vivo (Figure S7). To further confirm the role of *LlERF092* in heat stress response, the pot‐growth transgenic lily plants were used for the thermotolerance test. The results showed that LlERF092‐OE plants were thermotolerant compared to WT (Figure 3G). Moreover, the RIL was lower in LlERF092‐OE plants than in WT after heat stress (Figure 3H). Compared to the WT plants, the LlERF092‐OE plants exhibited a higher accumulation of chlorophyll (Figure 3I,J) and carotenoid (Figure 3K) in leaves after heat stress. These results indicated that the overexpression of *LlERF092* enhanced thermotolerance in lily.

### 2.4 LlERF092 Interacts With LlETO1

**FIGURE 4:**
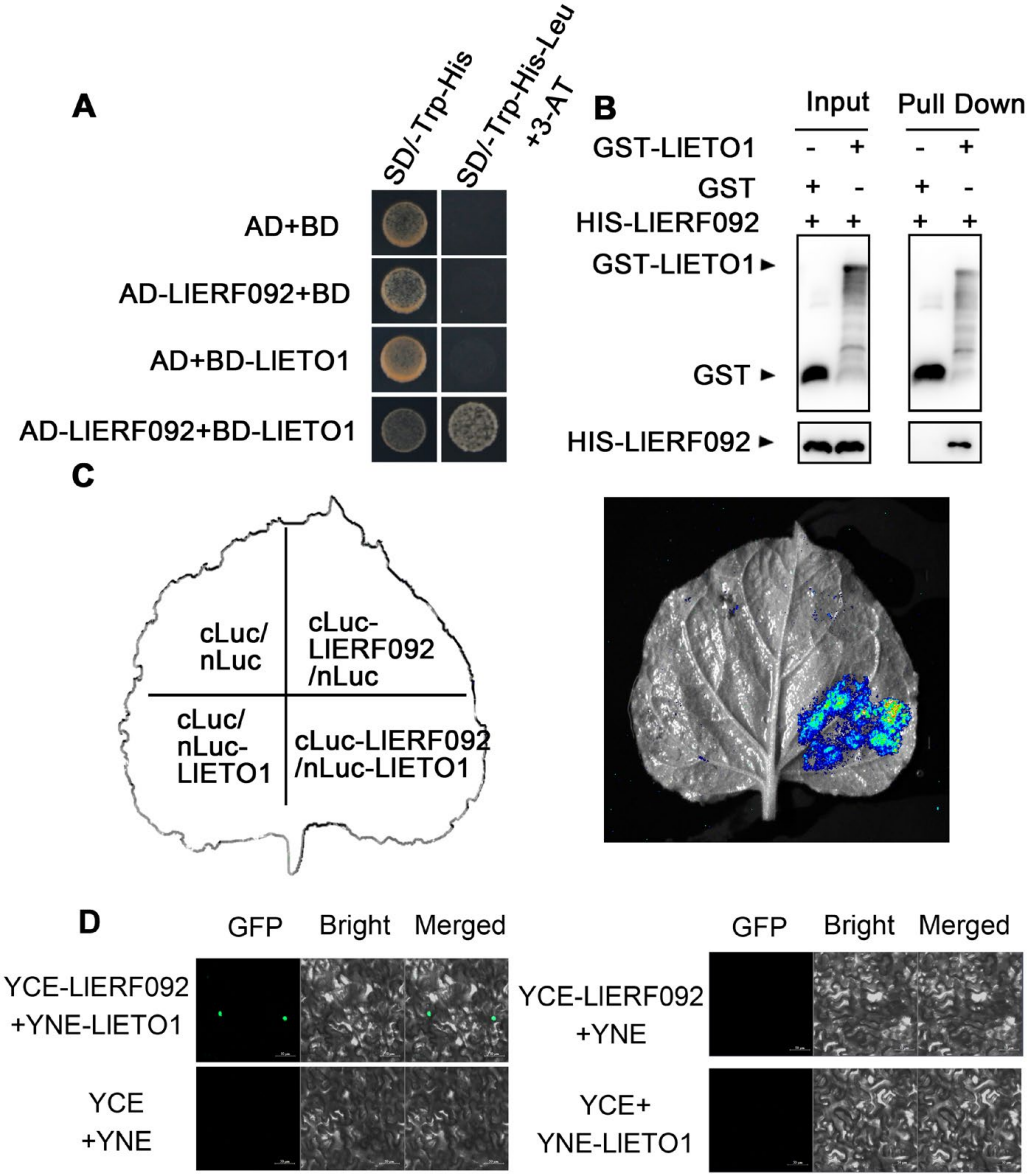
LlERF092 interacts with LlETO1. (A) LlERF092 interacted with LlETO1 in yeast. AD, pGADT7; BD, pGBKT7; SD, synthetic dropout medium. The representative picture came from three independent experiments. (B) GST‐Pull down analysis of LlERF092 and LlETO1 in vitro. ‘Input’ represented the protein mixtures before the experiment; ‘Pull down’ represented the purified protein mixture; the ‘+’ indicates addition; and the ‘−’ indicates a nonexistence. GST, glutathione S‐transferase; HIS, histidine. (C) LCI assay of LlERF092 and LlETO1 in *N. benthamiana* leaves. Representative image based on three independent experiments. (D) BiFC assay of LlERF092 and LlETO1 in *N. benthamiana* leaves. The representative image based on three independent experiments. GFP, green fluorescence protein; BF, the bright light channel images; Merged, the overlay plots. Scale bar = 50 μm.

To identify proteins that interact with LlERF092 under heat stress conditions, we conducted an in vitro pull‐down (IP) and mass spectrometry (MS) assay utilising LlERF092‐His protein. Nuclear proteins were extracted from the lily leaves and subsequently analysed using a histone H3 antibody, with target bands observed at an approximate molecular weight of 16 kDa (Figure S8A). The outlined pull‐down procedure is depicted in Figure S8B. SDS‐PAGE electrophoresis revealed that, following heat treatment, several unidentified bands exhibited a marked enhancement compared to the untreated samples (Figure S8C). Mass spectrometry identified a total of 64 protein‐coding genes with the potential to interact with LlERF092 (Table S3). Since ERF092 and MBF1c have been reported to participate in the ethylene signalling pathway, we focused on two proteins implicated in ethylene‐regulated pathways: EER5 (enhanced ethylene response protein 5‐like) and ETO1 (ethylene‐overproduction protein 1). Y2H assays confirmed an interaction between LlETO1 and LlERF092 (Figure S9). The ORF of *LlETO1* was found to be 1980 bp, predicting a protein of 659 amino acids. Transcript levels of *LlETO1* increased rapidly at 20 min after the heat treatment (Figure S10), paralleling the expression trend of *LlERF092*. LlETO1 was localised in both the nucleus and cytoplasm (Figure S11). Further Y2H analysis indicated that LlETO1 interacted with LlERF092 (Figure 4A). Furthermore, GST‐pull down assays demonstrated a direct interaction between LlERF092 and LlETO1 proteins in vitro (Figure 4B). The LCI assay showed the LUC signals in the region of *N. benthamiana* leaves co‐expressing *LlERF092* and *LlETO1*, indicating a protein interaction in vivo (Figure 4C). Additionally, the BiFC assay confirmed that LlERF092 interacted with LlETO1, with the interaction localised to the nucleus (Figure 4D), consistent with the observed nuclear localization of LlERF092 (Figure S4), and LlETO1 also had nuclear localization (Figure S11). Collectively, these findings suggested that LlERF092 physically interacted with LlETO1.

### 2.5 LlETO1 Cooperates With LlERF092 to Improve Thermotolerance In Vivo

**FIGURE 5:**
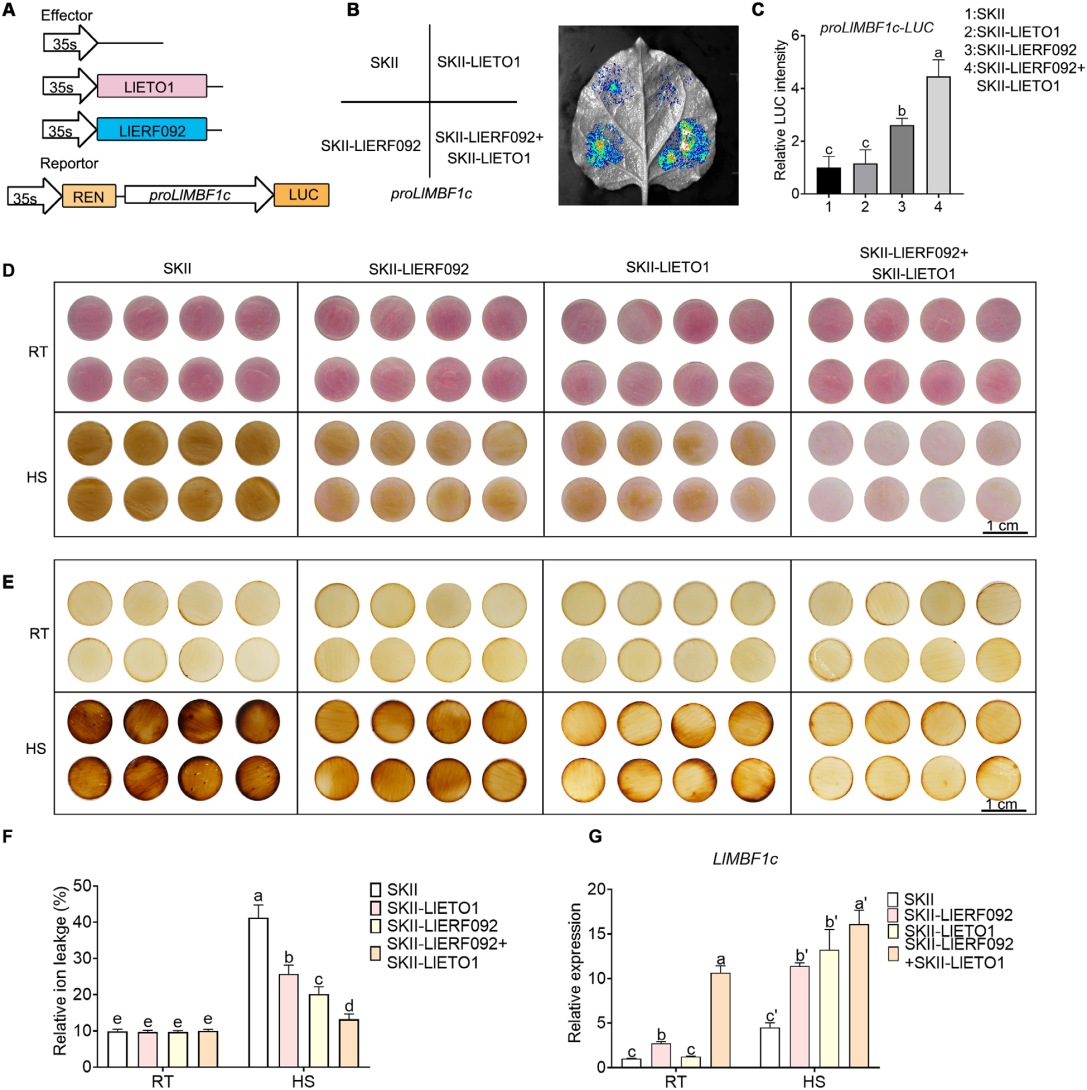
LlETO1 cooperates with LlERF092 in thermotolerance. (A) Pattern diagram of vector construction. (B) LUC signal analysis in the *N. benthamiana* leaves. Representative image from three independent experiments. (C) Measurement of the relative fluorescence intensity in (B). Data are shown as the mean ± SD of three replicates, with different letters indicating statistically significant difference (Student–Newman–Keuls test, *p* < 0.05). (D) Phenotype analysis of petal discs overexpressing *LlERF092* and *LlETO1*. RT, cultured at 22°C; HS, cultured at 40°C. Scale bar = 1 cm. (E) DAB staining of petal discs overexpressing *LlERF092* and *LlETO1*. RT, cultured at 22°C; HS, cultured at 40°C. Scale bar = 1 cm. (F) Relative ion leakage analysis of petal discs overexpressing *LlERF092* and *LlETO1*. RT, cultured at 22°C; HS, cultured at 40°C. Data are shown as the mean ± SD of three replicates, with different letters indicating statistically significant difference (Student–Newman–Keuls test, *p* < 0.05). (G) Relative expression of *LlMBF1c* in transient overexpression petal discs. RT, cultured at 22°C; HS, cultured at 40°C. 18s rRNA of lily served as the reference gene. Data are shown as the mean ± SD of three replicates, with different letters indicating statistically significant difference (Student–Newman–Keuls test, *p* < 0.05).

To investigate whether the interaction between LlERF092 and LlETO1 affects the expression of *LlMBF1c*, we conducted a dual‐luciferase reporter assay. The results demonstrated that the promoter activity of *LlMBF1c* was activated by LlERF092; this activity was further enhanced when *LlETO1* was co‐expressed with *LlERF092* (Figure 5A–C). To assess whether LlETO1 binds to the promoter of *LlMBF1c*, we performed a Y1H assay; the results indicated that LlETO1 did not bind to the promoter of *LlMBF1c* (Figure S12). Compared to petal discs overexpressing either *LlERF092* or *LlETO1*, those co‐overexpressing both genes exhibited less discoloration (Figure 5D). Additionally, no significant difference in H_2_O_2_ accumulation was observed under normal conditions (Figure 5E). After heat stress, compared to the *LlERF092* or *LlETO1* overexpressing petal discs, the *LlERF092* and *LlETO1* co‐overexpressing petal discs showed a lower H_2_O_2_ accumulation (Figure 5E). The RIL of co‐overexpressing petal discs was the lowest among the test groups (Figure 5F); the co‐overexpressing petal discs also showed a further increased accumulation of *LlMBF1c* compared to separate *LlERF092* overexpression (Figure 5G).

**FIGURE 6:**
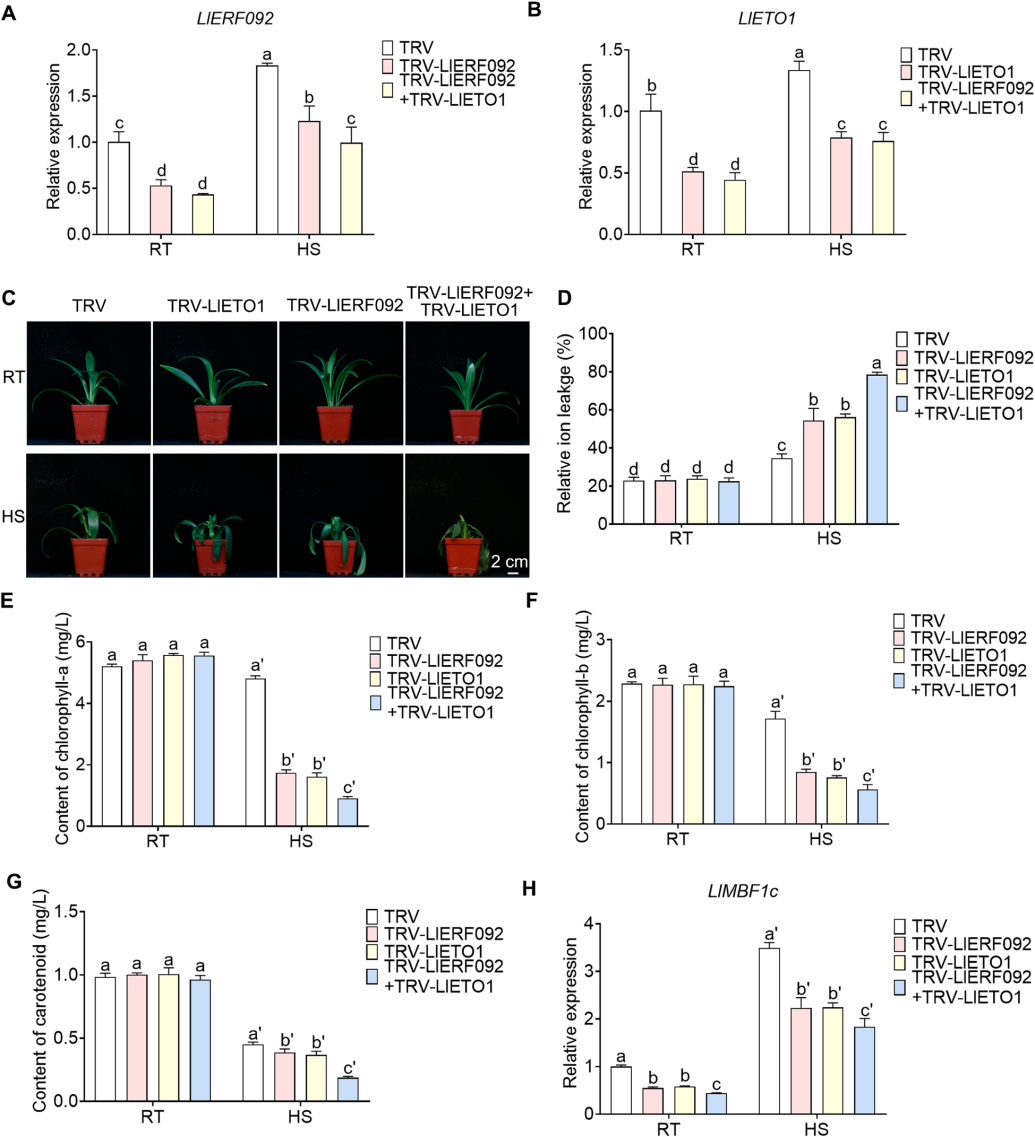
Co‐silencing of *LlETO1* and *LlERF092* reduces thermotolerance of lily. (A) Expression analysis of *LlERF092* in gene‐silenced lily plants. RT, room temperature, cultured at 22°C; HS, cultured at 45°C. 18s rRNA of lily served as the reference gene. Data are shown as the mean ± SD of three replicates, with different letters indicating statistically significant difference (Student–Newman–Keuls test, *p* < 0.05). (B) Expression analysis of *LlETO1* in gene‐silenced lily plants. RT, room temperature, cultured at 22°C; HS, cultured at 45°C. 18s rRNA of lily served as the reference gene. Data are shown as the mean ± SD of three replicates, with different letters indicating statistically significant difference (Student–Newman–Keuls test, *p* < 0.05). (C) Phenotype analysis of gene‐silenced plants before and after heat stress. Lily bulbs with a diameter of 1.5 cm were cultured for 5 weeks. RT, room temperature, 22°C; HS, 45°C, recovery 4 days at 22°C. Scale bar = 2 cm. (D) Relative ion leakage analysis of gene‐silenced plants. RT, room temperature, cultured at 22°C; HS, cultured at 45°C. Data are shown as the mean ± SD of three replicates, with different letters indicating statistically significant difference (Student–Newman–Keuls test, *p* < 0.05). (E–G) Chlorophyll a (E), chlorophyll b (F), and carotenoid (G) content analysis of lily plants. RT, room temperature, cultured at 22°C; HS, cultured at 45°C. Data are shown as the mean ± SD of three replicates, with different letters indicating statistically significant difference (Student–Newman–Keuls test, *p* < 0.05). (H) Relative expression of *LlMBF1c* in gene‐silenced lily plants. RT, room temperature, cultured at 22°C; HS, cultured at 45°C. 18s rRNA of lily served as the reference gene. Data are shown as the mean ± SD of three replicates, with different letters indicating statistically significant difference (Student–Newman–Keuls test, *p* < 0.05).

Additionally, the interaction effect between LlERF092 and LlETO1 was also analysed in lily with the tobacco rattle virus (TRV)‐induced gene silencing (TRV‐VIGS) method. Expression analysis revealed that *LlERF092* accumulation was reduced in both *LlERF092*‐silenced and co‐silenced plants (Figure 6A), while *LlETO1* accumulation was similarly decreased in *LlETO1*‐silenced and co‐silenced plants (Figure 6B). Compared to the TRV control, the *LlERF092*‐silenced, *LlETO1*‐silenced, and co‐silenced plants showed heat sensitivity (Figure 6C); silencing of *LlERF092* or *LlETO1* in lily plants increased the RIL of leaves after heat treatment, suggesting that silencing *LlERF092* or *LlETO1* impaired the thermotolerance of lily. However, compared to silencing LlERF092 or LlETO1, co‐silencing *LlERF092* and *LlETO1* exhibited a higher RIL after heat treatment, indicating that co‐silencing them exacerbated the heat damage to lily leaves (Figure 6D). Compared to the TRV control, silencing of *LlERF092* or *LlETO1* reduced the accumulation of chlorophyll and carotenoid in lily leaves after heat treatment (Figure 6E–G), indicating that silencing *LlERF092* or *LlETO1* damaged the photosynthetic system of lily. Co‐silencing of *LlERF092* and *LlETO1* further resulted in a lower accumulation of chlorophyll and carotenoid after heat treatment, suggesting that co‐silencing exacerbated the heat damage to the photosynthetic system of lily leaves (Figure 6E–G). Expression analysis of *LlMBF1c* revealed that silencing *LlERF092* resulted in a decrease in *LlMBF1c* expression, with co‐silencing *LlERF092* and *LlETO1* further reducing its expression (Figure 6H). These results suggested that both *LlERF092* and *LlETO1* positively regulated thermotolerance in lily, and their interaction enhanced this trait. Collectively, the findings indicate that LlETO1 works synergistically with LlERF092 to regulate thermotolerance.

### 2.6 The Heat‐Responsive Role of LlERF092/LlETO1‐LlMBF1c Module Depends on the Ethylene Signal Pathway

**FIGURE 7:**
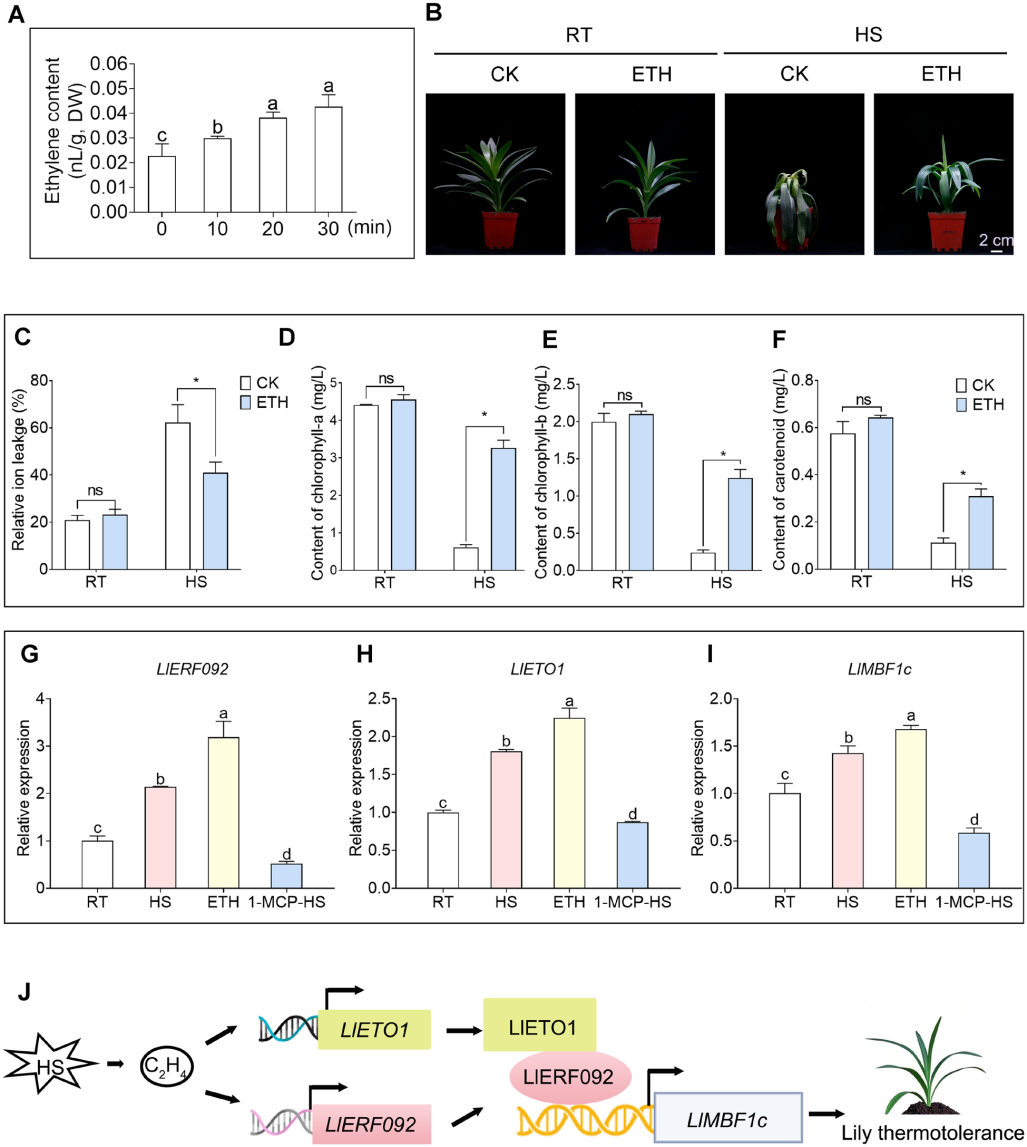
Ethylene is involved in lily thermotolerance. (A) Ethylene content analysis under heat stress. Data are shown as the mean ± SD of three replicates, with different letters indicating statistically significant difference (Student–Newman–Keuls test, *p* < 0.05). DW represented dry weight. (B) Phenotype analysis of ethephon‐treated lily plants. Lily bulbs with a diameter of 1.5 cm were cultured for 10 weeks. A total of 16 lily plants were treated with ethephon, of which 13 showed more severe leaf burning and wilting compared to the untreated plants. RT, room temperature, cultured at 22°C; HS, cultured at 45°C for 12 h, followed by a 4‐day recovery period at 22°C. Scale bar = 2 cm. (C) Determination of relative ion leakage of ethephon‐treated lily plants before and after heat stress. ns represented no significant difference; the data are shown as the mean ± SD of three replicates (Student's *t*‐test, *p < 0.05). (D–F) Determination of chlorophyll a (D), chlorophyll b (E), and carotenoid (F) content of ethephon‐treated lily plants. RT, room temperature, cultured at 22°C; HS, cultured at 45°C. Data are shown as the mean ± SD of three replicates (Student's *t*‐test, *p < 0.05). (G–I) Expression analysis of *LlERF092* (G), *LlETO1* (H), and *LlMBF1c* (I) with different treatments. HS, heat treatment at 37°C for 30 min; ETH, 2 ppm ethephon treatment for 30 min; 1‐MCP‐HS, 1‐MCP treated for 3 h and then treated with 37°C for 30 min. The lily plants cultured at 22°C served as control; 18s rRNA served as the reference gene. Data are shown as the mean ± SD of three replicates, with different letters indicating statistically significant difference (Student–Newman–Keuls test, *p* < 0.05). (J) A simple model of the LlETO1‐LlERF092‐LlMBF1c module improves thermotolerance under heat stress. Under high‐temperature conditions, plants produce ethylene, which induces the expression of *LlERF092* and *LlETO1*, LlERF092 binds to the promoter of *LlMBF1c* and activates its expression for improving lily thermotolerance. LlETO1 interacts with LlERF092 in the nucleus and synergizes with LlERF092 to regulate *LlMBF1c* and further improve lily thermotolerance. The pointed–end arrows indicate positive effects.

To investigate the relationship between ethylene and thermotolerance in lily, we examined ethylene production in response to heat stress. It was found that ethylene production increased gradually within 30 min of heat treatment (Figure 7A). Notably, lily plants pre‐treated with 2 ppm ethephon (ETH) for 3 h displayed a healthy green colour, in contrast to control plants, which exhibited signs of burning and wilting (Figure 7B). While ethephon treatment did not affect relative ion leakage under normal conditions, control plants showed higher relative ion leakage after heat treatment compared to those treated with ethephon (Figure 7C). Additionally, there were no significant differences in the contents of chlorophyll a, chlorophyll b, or carotenoids between ethylene‐treated plants and controls under normal conditions. However, after heat stress, the levels of chlorophyll a, chlorophyll b, and carotenoids were higher in plants treated with ethephon (Figure 7D–F). The expression analysis indicated that the expression levels of *LlETO1*, *LlERF092*, and *LlMBF1c* were induced by both ethephon treatment and heat stress (Figure 7G–I). Interestingly, when plants were treated with the ethylene synthesis inhibitor 1‐methylcyclopropene (1‐MCP) followed by heat stress, the expression of *LlETO1*, *LlERF092*, and *LlMBF1c* decreased compared to plants exposed directly to heat stress without inhibition. These results suggested that the heat‐responsive role of the LlERF092/LlETO1‐LlMBF1c module is dependent on the ethylene signal pathway.

## 3 Discussion

Although many studies have highlighted the crucial roles of ethylene and ethylene signalling components in plant responses to various stresses (Huang et al. 2021; Zhang et al. 2023; Li, Wu, et al. 2022; Li, Zhou, et al. 2022), the specific role of ethylene in heat stress response has received comparatively less attention. Our study identified two interacting ethylene signalling components, LlERF092 and LlETO1, and demonstrated that their interaction enhanced the activation of LlERF092 to the promoter of *LlMBF1c*, thus positively regulating thermotolerance (Figure 7J).

MBF1c has emerged as a key regulator of thermotolerance in various species and is induced by heat stress, including Arabidopsis, *Triticum aestivum* , and *Cucumis sativus* (Suzuki et al. 2008; Qin et al. 2015; Tian et al. 2022). In our previous study, we also found that *LlMBF1c* is rapidly induced by heat stress (Xiang et al. 2024). Most studies have focused on the downstream regulators of MBF1c (Tian et al. 2022; Liu et al. 2023). In this study, we identified that the promoter F2 fragment of *LlMBF1c* is a core element responsible for heat stress (Figure 1).

ERF092 (also known as ERF1) is a core regulator in the ethylene signalling pathway (Solano et al. 1998). In other studies, AtERF1‐overexpressing lines were more tolerant to drought and salt stress, and it also can delay floral transition and inhibit lateral root emergence (Cheng et al. 2013; Chen et al. 2021; Zhao et al. 2023). In rice, ERF1 has been identified as a negative regulator of grain filling and involved in gibberellin‐mediated seedling establishment (Schmidt et al. 2014). These data suggested that ERF1 may be widely involved in biotic and abiotic stress. In our study, we focused on the AP2/ERF family member LlERF092. Overexpression of *LlERF092* increased the thermotolerance of lily, and systematic silencing of *LlERF092* decreased the thermotolerance of lily (Figure 6), suggesting that LlERF092 plays a positive role in thermotolerance. ERF1 positively regulates the defence responses to *Rhizoctonia cerealis* and freezing stresses by activating defence‐related genes and stress‐related genes downstream of the ethylene signalling pathway (1‐AMINOCYCLOPROPANE‐1‐CARBOXYLIC ACID OXIDASE2 [ACO2]) (Zhu et al. 2014). ERF1 regulates lateral root emergence by activating *PIN‐FORMED 1* (*PIN1*) and *Auxin Response Factor 7* (*ARF7*) (Zhang et al. 2023). Those results suggested that ERF1 functions as a transcription factor by activating downstream genes. Similarly, we found that LlERF092 has transcription activity (Figure S5), and it bound to the heat‐induced fragment of the *LlMBF1c* promoter and activated *LlMBF1c* expression (Figure 2). Both LlERF110 and LlERF012 are members of the ERF family and play roles in the thermotolerance of lily (Li, Wu, et al. 2022; Li, Zhou, et al. 2022; Li et al. 2024). The expression of *LlERF012* and *LlERF110* was increased under ethephon treatment (Figure S13A,B), which suggested that ethylene induced their expression. However, Y1H assays showed that either LlERF012 or LlERF110 did not bind to the promoter of *LlMBF1c* (Figure S13C). These findings suggested that LlERF012 and LlERF110 might enhance the thermotolerance of lily not by directly regulating *LlMBF1c*, but by modulating the HSF‐mediated pathway (Li et al. 2024). Additionally, LlERF110 may be involved in the ROS‐mediated pathway (Li, Wu, et al. 2022; Li, Zhou, et al. 2022). These results suggest that the LlERF092 increase in thermotolerance may be due to increasing the expression of *LlMBF1c* under heat stress (Figure 2). ETHYLENE OVERPRODUCER 1 (ETO1) is a plant‐specific protein family characterised by the presence of a Broad‐complex, Tramtrack, Bric‐a‐brac (BTB) domain and seven tetratricopeptide repeat (TPR) motifs (Chae and Kieber 2005). ETO1 plays a pivotal regulatory role in various aspects of plant development, including lateral root formation, grain filling, spikelet fertility, and elongation, by modulating ethylene signalling (Du et al. 2014; Gu et al. 2021; Cheng et al. 2024). These results suggest that ETO1 is one of the most important regulators of ET signalling during plant development. In rice, ETOL1 inhibited the transportation of carbohydrates from leaves to seeds, leading to reduced grain filling and spikelet fertility during the reproductive stage under drought stress. Conversely, under submerged environments, ETOL1 promoted carbohydrate utilisation and energy production, facilitating the growth of top leaves above water levels (Du et al. 2014). Additionally, ETO1 promotes root hair growth under nitrogen, phosphorus, or potassium deficiency by upregulating ethylene‐specific genes, including EIL2, ROOT HAIR SPECIFIC 13 (RHS13), and SHORT HYPOCOTYL UNDER BLUE 1 (SHB1) (Cheng et al. 2024). These findings suggest that ETO1 can exert both positive and negative regulatory effects in response to abiotic stress. In the shoot apical meristem (SAM) of Arabidopsis, ETO1 and EOL1 were induced by heat stress and ethylene, and *AtETO1* and *AtEOL1* functioned downstream of the HS memory gene HSFA7b, suggesting their involvement in the plant heat shock response (HSR) through the HSF‐mediated pathway (John et al. 2024). Herein, we found that LlETO1 positively regulates thermotolerance (Figures 5 and 6). Its mRNA levels increased significantly under heat stress (Figure S10). Interestingly, we found that LlETO1 plays a role in plant HSR through a distinct mechanism independent of the HSF‐mediated pathway. Instead, LlETO1 interacts with LlERF092, an ethylene signalling response factor, to promote the activation of *LlMBF1c* (Figures 4 and 6). Moreover, co‐overexpression of *LlETO1* and *LlERF092* increased the accumulation of *LlMBF1c* (Figure 5). Based on these results, we propose that LlETO1 enhances plant thermotolerance by interacting with LlERF092, thereby upregulating *LlMBF1c* expression in response to heat stress.

Exogenous application of ethephon significantly enhanced the thermotolerance of lily (Figure 7) indicating that ethylene plays a positive regulatory role in its thermotolerance. This finding aligns with the previous studies in *Solanum lycopersicum* , *Arabidopsis thaliana* , and *Triticum aestivum* (Larkindale et al. 2005; Wu and Yang 2019; Yamauchi et al. 2014), further supporting the conserved role of ethylene in heat stress responses across plant species. The expression levels of *LlERF092* and *LlETO1* were upregulated under both heat stress and ethephon treatment (Figures 3, 7; Figure S10), suggesting that their transcription is regulated by elevated temperatures and ethylene signalling. Furthermore, pretreatment with 1‐MCP, an ethylene action inhibitor, reduced the mRNA levels of *LlERF092* and *LlETO1* compared to heat stress treatment alone. This observation, coupled with the increased ethylene release under heat stress conditions (Figure 7), strongly indicates that *LlERF092* and *LlETO1* function downstream of ethylene signalling. Based on these results, we propose that ethylene‐mediated induction of *LlERF092* and *LlETO1* is a key mechanism underlying lily's thermotolerance. This regulatory pathway highlights the intricate interplay between ethylene signalling and heat stress responses in plants. Therefore, we speculated that LlERF092 and LlETO1 functioned downstream of ethylene.

Arabidopsis pretreatment with ethylene, abscisic acid (ABA), and salicylic acid (SA) increases thermotolerance (Huang et al. 2021). However, the molecular connections between ethylene and other hormones under heat stress are sparse. In Arabidopsis, *ERF092* is downstream of EIN2 and EIN3, participating in the ethylene signalling cascade. Its constitutive expression phenocopies ethylene overproduction (Solano et al. 1998). ERF1 is also regulated by various phytohormones including ABA and SA, ethylene, and auxin, suggesting that ERF1 is at the cross‐roads of multiple hormone signalling pathways (Zhang et al. 2023; Li, Wu, et al. 2022; Li, Zhou, et al. 2022; Makhloufi et al. 2014). MBF1cenhances thermotolerance through the ethylene and salicylic acid (SA) signalling pathways (Suzuki et al. 2008). ABA plays an essential role in the establishment of drought tolerance mediated by MBF1c (Zandalinas et al. 2016). In this study, we found that both LlERF092 and LlMBF1c are involved in regulating thermotolerance through the ethylene signalling pathway. However, whether LlERF092 also contributes to the heat stress response by modulating other hormonal pathways remains to be investigated in the future.

In summary, we propose a transcriptional regulatory module comprising LlERF092, LlETO1, and LlMBF1c that functions in a cascade to mediate ethylene‐associated thermotolerance in lily (Figure 7J). This study provides novel insights into the molecular mechanisms underlying ethylene‐enhanced plant thermotolerance, and offers a valuable theoretical foundation for the development of new lily varieties with high thermotolerance through molecular breeding techniques.

## 4 Materials and Methods

### 4.1 Plant Materials

Tissue‐cultured seedlings of ‘White Heaven’ ( *L. longiflorum* ) were grown in a Murashige and Skoog medium (MS). The seeds of *Nicotiana benthamiana* (*N. benthamiana*) were immersed in water at a temperature of 4°C for a period of 3 days, after which they were sown and cultivated on MS for a duration of 10 days. Then, the seedlings of *N. benthamiana* were then moved to plastic containers. Every plant material was cultivated in controlled conditions at 22°C during the day (16 h) and 16°C at night (8 h).

### 4.2 Generation of Transgenic Lines and Plant Transformation

To generate overexpressing transgenic lines, the coding region of *LlERF092* was inserted into pCAMBIA1300‐C‐GFP (Abcam, Cambridge, UK). The LlERF092‐GFP plasmid was transformed into *Agrobacterium tumefaciens* EHA105. The *Agrobacterium tumefaciens* containing LlERF092‐GFP was infiltrated into the lily callus according to Song's protocol (Song et al. 2020). Briefly, bacteria were cultured in 10 mL LB liquid medium to a density of OD_600_ = 1.0 and re‐cultured in 100 mL LB liquid medium to OD_600_ = 0.6. Then, the bacteria were resuspended in MS liquid medium for 2 h. The calli were then immersed in MS liquid medium for 10 min. Finally, the calli were grown on co‐culture medium (MS + 1.0 mg/L Picloram +0.2 mg/L NAA + 100 μM AS) for 2–3 days and then transferred to the screening medium (MS + 1.0 mg/L Picloram +0.2 mg/L NAA + 30 mg/L Hyg) for 1 month and cultured on MS medium containing 0.5 mg/L 6‐BA. The PCR amplification, western blot, and quantitative PCR (qPCR) were used to confirm the successful transformation of the lily plants. Transgenic lines and wild‐type plants were subjected to heat stress in a light‐controlled growth incubator (LianCe, GZL‐P80‐A, China) at 45°C for 12 h, followed by a recovery period at 22°C under normal growth conditions. Phenotype observations were conducted at 4 days post‐treatment.

### 4.3 Gene and Promoter Isolation

Based on the transcriptome data, we cloned the genes *LlERF092* and *LlETO1* from the ‘White heaven’ (Wu, Li, et al. 2024). The promoters of *LlMBF1c* and *LlERF092* were isolated from the ‘White Heaven’ genome using the fusion primer and nested integrated PCR (FNIP‐PCR) method (Aboul and Oraby 2019; Wang et al. 2011; Xu et al. 2024). The specific primer sequences have been catalogued in Table S1.

### 4.4 Gene Expression Assay

The lily ‘White Heaven’ tissue culture seedlings were used to detect the gene expression. The seedlings were exposed to 37°C for different intervals: 0, 10, 20, 30, and 60 min for heat stress treatments. For ethephon treatment, tissue‐cultured seedlings were treated with 2 ppm ethephon in a closed container. For 1‐methylcyclopropene (1‐MCP) treatment, tissue‐cultured seedlings were treated with 2 ppm 1‐MCP for 3 h, followed by exposure to 37°C in a light‐controlled incubator (LianCe, GZL‐P80‐A, China) for 30 min. Subsequent to these treatments, total RNA was extracted from the leaves. The synthesis of cDNA was conducted using the R323‐01 kit (Vazyme, Nanjing, China). Quantitative PCR was used to measure the relative expression levels, and the 2^−ΔΔCT^ method was used to analyse the data (Livak and Schmittgen 2001). The 18s rRNA of the lily was used as the reference control.

### 4.5 Yeast One‐Hybrid Library Screening Assay

The Y1H library screening assays were conducted in line with the manufacturer's instructions (Clontech Laboratories). Briefly, the F2 fragment of *LlMBF1c* promoter was cloned into pHIS2.1 (Clontech). The pGADT7 empty vector and pHIS2.1‐LlMBF1c‐F2 plasmids were introduced into Y187 and cultured on synthetic defined (SD) medium without tryptophan, leucine, and histidine (SD/‐Trp‐Leu‐His). 3‐amino‐1,2,4‐triazole (3‐AT) was added to the SD/‐Trp‐Leu‐His medium to determine optimal inhibitory concentration. The plasmid library of ‘White Heaven’ and pHIS2.1‐LlMBF1c‐F2 plasmid were transformed to yeast Y187 and plated on SD/‐Trp‐Leu‐His medium with 3‐AT. The grown clones were picked for PCR and sequenced. In the one‐to‐one validation assay, the pGADT7‐gene and pHIS2.1‐LlMBF1c‐F2 plasmids were co‐transformed into yeast Y187 and cultured on SD/‐Trp‐Leu‐His medium with 3‐AT for 3 days.

### 4.6 Yeast One‐Hybrid (Y1H) Assay

The coding sequence of *LlETO1* was cloned into the pJG (Clontech) vector, and the promoter regions of *LlMBF1c* were inserted into the pLacZi (Clontech) vector. Both vectors were then introduced into the yeast EGY48 strain and incubated at 30°C for 3 days on SD/‐Trp‐Ura medium. The protein‐DNA interactions were investigated on SD/‐Trp‐Ura medium supplemented with X‐Gal.

### 4.7 Chromatin Immunoprecipitation (ChIP)‐qPCR Assay

For ChIP assay in transient overexpression lily calli, the 2 g of callus that harboured either 1300‐GFP empty plasmid or 1300‐LlERF092‐GFP plasmid were ground into fine powder, respectively (Wu, Gong, et al. 2024). For ChIP assay in stable transgenic lily plants, the 5 g of leaves from wild‐type and *LlERF092‐GFP* transgenic plants were ground into fine powder, respectively. The powder was then solubilised in a solution containing 10 mM Tris–HCl at pH = 8.0, 0.4 M sucrose, 1 mM MgCl_2_, 1 mM CaCl_2_, 1% Triton X‐100, 1 mM DL‐dithiothreitol (DTT), 0.1 mM PMSF, protease inhibitor cocktail, and 1% formaldehyde. The mixture was filtered through two layers of Miracloth (Calbiochem) and centrifuged to remove the supernatant. The sediment was then resuspended in solution 2 with 10 mM Tris–HCl at pH = 8.0, 0.25 M sucrose, 10 mM MgCl_2_, 1% Triton X‐100, 1 mM DTT, 0.1 mM PMSF, and protease inhibitor cocktail and centrifuged to remove the supernatant. The sediment was dissolved with solution 3 (10 mM Tris–HCl at pH = 8.0, 1.7 M sucrose, 2 mM MgCl_2_, 0.15% Triton X‐100, 1 mM DTT, 0.1 mM PMSF, and protease inhibitor cocktail) and centrifuged to remove the supernatant. Subsequently, the sediment was lysed in a buffer optimised for releasing nuclear proteins and chromatin, composed of a Tris–HCl, EDTA, and SDS cocktail. The chromatin was then interrupted by an ultrasonic crusher. The sonicated chromatin (100 μL) was mixed with A/G beads for 12 h at 4°C and washed with washing buffer 1 (20 mM Tris–HCl at pH = 8.0, 150 mM NaCl, 2 mM EDTA, 0.1% SDS, 1% Triton X‐100), and washing buffer 2 (20 mM Tris–HCl at pH = 8.0, 500 mM NaCl, 2 mM EDTA, 0.1% SDS, 1% Triton X‐100). Then the washed beads were boiled in elution buffer (50 mM Tris–HCl, pH 8.0, 10 mM EDTA, 1% SDS). Post reverse‐crosslinking, the DNA was extracted using the QIAquick PCR Purification Kit supplied by Qiagen. Quantitative PCR (qPCR) analysis was performed to analyse the enrichment of the target sequence within the samples.

### 4.8 Dual—Luciferase Reporter Assay

The *LlMBF1c* promoter was inserted into the pGreenII0800‐LUC vector. The open reading frame of *LlERF092* and *LlETO1* was individually cloned into the pGreenII62‐SK plasmid, resulting in the creation of effector vectors. Each of these vectors was then transformed into *A. tumefaciens* strain GV3101 (pSoup). Thereafter, bacteria harbouring the reporter and effector vectors were infiltrated into the *N. benthamiana* leaves for LUC activity observation. Fluorescence signals were observed after 48 h.

### 4.9 Promoter Activity Detection

Promoter activity detection was performed following the method described by Ding and colleagues with slight alterations (Ding et al. 2024). The *LlMBF1c* promoter and its truncated fragments, designated D1, D2, D3, and D4, and the *LlERF092* promoter were constructed into pCAMBIA1391. The recombinant plasmids were transferred into the *A. tumefaciens* strain GV3101. For transient transformation, the inner petals of lily ‘Sorbonne’ were separated as 1 cm‐diameter discs by a hole puncher. Petal discs were infiltrated with *A. tumefaciens* using a vacuum for 15 min. Then, the petal discs were incubated at 22°C in a dark environment for 24 h, and then exposed to light for 24 h before 37°C treatment in a light‐controlled incubator (LianCe, GZL‐P80‐A, China). The petal discs were then immersed in GUS (β‐glucuronidase) staining solution and vacuum infiltrated for 10 min and incubated in dark conditions for 8 h. Then, the staining solution was removed, and the petal discs were decolorized with 70% ethanol.

### 4.10 Yeast Two‐Hybrid (Y2H) Assay

The ORF of *LlERF092* was cloned into the pGADT7 vector, while the ORF of *LlETO1* was inserted into the pGBKT7 vector. Both vectors were introduced into the yeast AH109 strain; the empty pGADT7 and pGBKT7 vectors served as negative controls. Following transformation, the transformed yeast was then incubated on SD medium lacking Leu and Trp, and further assays were conducted on SD medium lacking Leu, Trp, and His with or without 3‐AT.

### 4.11 In Vitro Pull‐Down (IP) and Mass Spectrometry (MS) Assay

The ORF of *LlERF092* was cloned into the pET32a vector, which was then introduced into *Escherichia coli* strain BL21. The LlERF092‐His protein was induced by adding isopropyl β‐D‐thiogalactoside (IPTG, 0.2 mM), following which it was purified and resuspended in phosphate buffer saline (PBS). The nuclear proteins of ‘White Heaven’ either treated at 37°C (LianCe, GZL‐P80‐A, China) for 1 h or untreated ‘White Heaven’ were extracted, respectively. Then, LlERF092‐His was incubated with nuclear proteins for 8 h at 4°C. Subsequent purification of LlERF092‐His and its interacting proteins was conducted using HIS magnetic beads, followed by separation of the proteins through SDS‐PAGE. Protein bands were excised and subjected to MALDI‐TOF analysis.

### 4.12 Electrophoretic Mobility Shift Assay (EMSA)

Purified LlERF092‐His protein was used for the electrophoretic mobility shift assay. The 3 μL of purified protein and 100 nM probes were incubated at 25°C for 10 min. EMSAs were conducted using an EMSA Kit (20148; Thermo Scientific) according to the manufacturer's instructions. The signal was detected by a CCD camera (Tannon, China).

### 4.13 GST‐Pull Down Assay

The coding sequence of *LlETO1* was cloned into the pGEX‐4T‐1 plasmid, yielding a fusion protein tagged with GST, named GST‐LlETO1. The proteins were purified by related magnetic beads. The purified HIS‐LlERF092 and GST‐LlETO1 proteins were incubated for 3 h at 4°C. Following this incubation, the mixture was purified by GST magnetic beads, and the purified mixture was detected by western blot with HIS and GST antibodies, respectively. GST protein acted as the negative control.

### 4.14 Bimolecular Fluorescence Complementation (BiFC) Assay

The ORF of *LlERF092* was inserted into the pSPYCE vector to form the YCE‐LlERF092 recombinant plasmid, and the coding region of *LlETO1* was cloned into the pSPYNE vector to form the YNE‐LlETO1 recombinant plasmid. The *A. tumefaciens* containing YCE‐LlERF092 and YNE‐LlETO1 plasmids were co‐infiltrated into *N. benthamiana* leaves, and the fluorescence signals were observed by a LSM800 microscope after 48 h.

### 4.15 Luciferase Complementation Imaging (LCI) Assay

For the LCI assay, the coding sequence of *LlERF092* was inserted into the pCAMBIA1300‐cLuc vector, and the ORF of *LlETO1* was inserted into the pCAMBIA1300‐nLuc vector (Chen et al. 2008). The *N. benthamiana* leaves were infiltrated with *A. tumefaciens* mixtures containing two recombinant plasmids. After infiltration, the *N. benthamiana* was incubated in the dark for 24 h, followed by light conditions for 24 h. Finally, a CCD camera (Tannon, China) was used for signal observation.

### 4.16 Subcellular Localization Analysis

The LlERF092‐GFP and LlETO1‐GFP constructs were transferred into the *A. tumefaciens* strain GV3101, respectively. Subsequently, the bacterial suspensions were infiltrated into the leaves of *N. benthamiana*. The plants underwent cultivation at 22°C for 2 days and were then subjected to a higher temperature of 37°C (30 min) in a light‐controlled incubator (LianCe, GZL‐P80‐A, China). The GFP fluorescence was observed with a Zeiss 800 confocal microscope.

### 4.17 Transient Overexpression Assay in Lily Petals

The SK‐II, SKII‐LlERF092, and SKII‐LlETO1 vectors were introduced into *A. tumefaciens* strain GV3101 (pSoup). The bacterial cultures were resuspended in the infiltration buffer and incubated in the dark for 5 h at 22°C. For transient overexpression, lily petal discs were obtained from a 10‐cm segment of unopened flower buds. Outer petals were removed, and 1‐cm diameter discs were excised from the inner petals using a hole punch. The discs were immersed in the bacterial solution and subjected to infiltration under a vacuum for 15 min. After infiltration, the discs were washed three times with deionised water and placed on a semi‐solid plate (0.4% agar) at 22°C for 72 h. For thermotolerance analysis, the discs were treated at 40°C and harvested immediately after 12 h to determine their relative ion leakage and perform DAB staining.

### 4.18 Virus‐Induced Gene Silencing in Lily Plants

The tobacco rattle virus (TRV)‐mediated gene silencing (TRV‐VIGS) method was employed to suppress gene expression in lilies. The *A. tumefaciens* carrying the TRV2‐gene and TRV2 plasmid were mixed with TRV1, respectively. For each silencing group, the 10 lily plants at the same growth stage were chosen for performing VIGS. The mixture was injected into the upper and middle leaves of the lily using a syringe. After injection, the plants were kept in the dark for 1 day, then transferred to normal growth conditions for 3 weeks. New leaves were harvested for gene expression analysis. At least 3 lily plants of each silencing group with lower gene expression were placed in a 45°C light‐controlled incubator for heat treatment, and phenotypic changes were observed and recorded at the 4 days of the recovery period.

### 4.19 Detection of Ethylene Production and Exogenous Ethephon Treatment

The leaves of ‘White Heaven’ were removed, weighed, and placed in a 30 mL closed bottle and incubated at 22°C for 6 h to avoid the contamination from wound‐induced ethylene release. Subsequently, the detached leaves were exposed to 37°C for different intervals: 0, 10, 20, and 30 min for ethylene levels analysis. The ‘White Heaven’ plants were treated with ethephon solution at a concentration of 2 ppm. The plants were then placed in airtight containers for 3 h. Then, the plants were subjected to 45°C for 12 h, the phenotype of the plants was continuously observed and recorded at 4 days after heat stress.

## Author Contributions

N.T. and Z.W. were responsible for the conception and design of the experiments; J.X. performed the experiments and wrote the first draft of the manuscript under the supervision of N.T., X.G., Q.F., L.D., Y.Z., S.X., T.L., and M.H. provided technical support; all authors consented to the final version of the manuscript.

## Conflicts of Interest

The authors declare no conflicts of interest.

## Supporting information




**Figure S1.** The self‐activation analysis of the F2 fragment from *LlMBF1c* promoter. SD, synthetic dropout medium; 3‐AT, 3‐amino‐1,2,4‐ triazole.
**Figure S2.** LlERF092 binds to the F2 fragment of the *LlMBF1c* promoter and activated its expression. (A) Growth status of transformed yeast strains on SD/‐Trp‐His‐Leu medium with varying concentrations of 3‐amino‐1,2,4‐triazole (3‐AT). SD, synthetic dropout medium. (B) LlERF092 activated the activity of the F2 fragment from the *LlMBF1c* promoter in *N. benthamiana*. (C) Measurement of the relative fluorescence intensity in (B). Data are shown as the mean ± SD of three replicates (Student’s *t*‐test, *p < 0.05).
**Figure S3.** Sequence analysis of LlERF092. (A) Phylogenetic analysis of LlERF092 and the members of the Arabidopsis ERF family. (B) Multiple comparisons of LlERF092 amino acid sequences. *Solanum lycopersicum* ERF1 (SlERF1); *Capsicum annuum* ERFLP1 (CaERFLP1); *Oryza sativa* EREBP1 (OsEREBP1); *Triticum aestivum* ERF1 (TaERF1); *Arabidopsis thaliana* ERF1(AtERF1); and *Lilium longiflorum* ERF092 (LlERF092).
**Figure S4.** Subcellular localization of LlERF092. RFP, the red fluorescence protein; BF, the bright light; GFP, green fluorescence protein; Merged, the overlay plots; RT, 22°C; HS, 37°C for 30 min. Scale Bars = 50 μm.
**Figure S5.** Transcriptional activity analysis in the yeast cells. (A) Schematic diagram of vector construction. (B) Analysis of the growth status of yeast cells. (C) Analysis of β‐galactosidase activity. GAL4 served as the positive control (CK+); BD was used as the negative control (CK−). Data are shown as the mean ± SD of three replicates, with different letters indicating statistically significant difference (Student–Newman–Keuls test, *p* < 0.05).
**Figure S6.** Identification of *LlERF092* overexpression lines. (A) Detection of *LlERF092* overexpression lines at DNA levels. PC, positive control, the LlERF092‐GFP plasmid served as positive control; WT, wild type. (B) Detection of *LlERF092* overexpression lines at protein levels. PC, positive control, GFP protein served as positive control; WT, wild type.
**Figure S7.** LlERF092 binds to the F2 fragment of the *LlMBF1c* promoter in *LlERF092‐*OE2 transgenic line. Data are presented as mean ± SD of three replicates (Student’s *t*‐test, *p < 0.05); ns, no significant difference.
**Figure S8.** IP‐MS screening of LlERF092 interaction proteins. (A) Immunoblotting assay of Histone 3 of nuclear protein extraction from ‘White Heaven’. (B) Schematic diagram of screening the LlERF092 interaction proteins by immunoprecipitation‐mass spectrometry technique; (C) SDS‐PAGE gel electrophoresis of HIS pull‐down assay of LlERF092 with nuclear proteins.
**Figure S9.** Yeast two‐hybrid assay of LlERF092 with LlETO1 and LlEER5. AD, pGADT7; BD, pGBKT7; SD, synthetic dropout medium. The representative picture came from three independent experiments.
**Figure S10.** Expression analysis of *LlETO1* under heat stress. The uniformly sized seedlings were treated with 37°C; 18s rRNA of lily served as a reference gene. Data are shown as the mean ± SD of three replicates, with different letters indicating statistically significant difference (Student–Newman–Keuls test, *p* < 0.05).
**Figure S11.** Subcellular localization of LlETO1. RFP, the red fluorescence protein; BF, the bright light; GFP, green fluorescence protein; Merged, the overlay plots. RT, 22°C; HS, 37°C for 30 min. Scale Bars = 50 μm.
**Figure S12.** Yeast one‐hybrid analysis relates to the binding of LlETO1 to the *LlMBF1c* promoter. (A) Growth status of transformed yeast cells on SD/‐Trp‐His‐Leu medium with different concentrations of 3‐amino‐1,2,4‐triazole (3‐AT). (B) Yeast one‐hybrid analysis of the interaction between LlETO1 and the *LlMBF1c* promoter. Yeast cell growth on SD/‐Ura‐Trp deficient medium containing X‐gal (5‐bromo‐4‐chloro‐3‐indolyl β‐d‐galactopyranoside) was used to assess this interaction. SD, synthetic dropout medium.
**Figure S13.** Expression analysis of *LlERF012* and *LlERF110* under ethephon treatment and yeast one‐hybrid assay of LlERF012 and LlERF110 interaction with the *LlMBF1c* promoter. (A) Expression analysis of *LlERF012* under ethephon treatment. 18s rRNA of lily served as the reference gene. Data are the mean ± SD of three replicates (Student’s *t*‐test, *p < 0.05). (B) Expression analysis of *LlERF110* under ethephon treatment. 18s rRNA of lily served as the reference gene. Data are the mean ± SD of three replicates (Student’s *t*‐test, *p < 0.05). (C) Growth status of transformed yeast cells on SD/−Trp‐His‐Leu medium with different concentrations of 3‐amino‐1,2,4‐ triazole (3‐AT). SD, synthetic dropout medium.

**Table S1.** Gene‐specific primers used in this study.
**Table S2.** Information on candidate factors with yeast one‐hybrid (Y1H) library screening.
**Table S3.** Information on candidate factors with IP‐MS.

## Data Availability

The data underlying this article are available from the corresponding author upon reasonable request.
