# Supplementary material for: An Ethylene‐Response Factor LlERF092 Coordinates With LlETO1 to Improve Thermotolerance by Activating LlMBF1c in Lily
Source: Plant Biotechnol J. 2025 Jul 22;23(11):4813–28. doi: 10.1111/pbi.70269 (PMC12576437; doi:10.1111/pbi.70269)
Supplement: Supplementary file 1 — Figure S1. The self‐activation analysis of the F2 fragment from LlMBF1c promoter. SD, synthetic dropout medium; 3‐AT, 3‐amino‐1,2,4‐ triazole. Figure S2. LlERF092 binds to the F2 fragment of the LlMBF1c promoter and activated its expression. (A) Growth status of transformed yeast strains on SD/‐Trp‐His‐Leu medium with varying concentrations of 3‐amino‐1,2,4‐triazole (3‐AT). SD, synthetic dropout medium. (B) LlERF092 activated the activity of the F2 fragment from the LlMBF1c promoter in N. benthamiana. (C) Measurement of the relative fluorescence intensity in (B). Data are shown as the mean ± SD of three replicates (Student’s t‐test, *p < 0.05). Figure S3. Sequence analysis of LlERF092. (A) Phylogenetic analysis of LlERF092 and the members of the Arabidopsis ERF family. (B) Multiple comparisons of LlERF092 amino acid sequences. Solanum lycopersicum ERF1 (SlERF1); Capsicum annuum ERFLP1 (CaERFLP1); Oryza sativa EREBP1 (OsEREBP1); Triticum aestivum ERF1 (TaERF1); Arabidopsis thaliana ERF1(AtERF1); and Lilium longiflorum ERF092 (LlERF092). Figure S4. Subcellular localization of LlERF092. RFP, the red fluorescence protein; BF, the bright light; GFP, green fluorescence protein; Merged, the overlay plots; RT, 22°C; HS, 37°C for 30 min. Scale Bars = 50 μm. Figure S5. Transcriptional activity analysis in the yeast cells. (A) Schematic diagram of vector construction. (B) Analysis of the growth status of yeast cells. (C) Analysis of β‐galactosidase activity. GAL4 served as the positive control (CK+); BD was used as the negative control (CK−). Data are shown as the mean ± SD of three replicates, with different letters indicating statistically significant difference (Student–Newman–Keuls test, p < 0.05). Figure S6. Identification of LlERF092 overexpression lines. (A) Detection of LlERF092 overexpression lines at DNA levels. PC, positive control, the LlERF092‐GFP plasmid served as positive control; WT, wild type. (B) Detection of LlERF092 overexpression lines at protein levels. P [file PBI-23-4813-s001.docx]

**Supplementary figures**

**
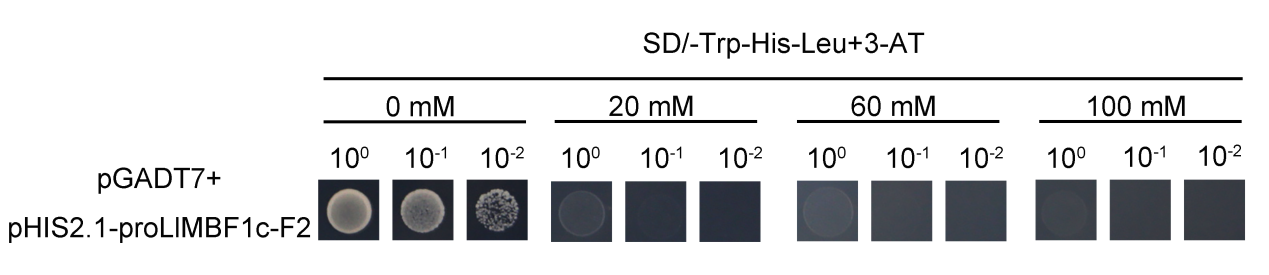
**

**Fig. S1.** The self-activation analysis of the F2 fragment from *LlMBF1c* promoter*.* SD, synthetic dropout medium; 3-AT, 3-amino-1,2,4- triazole.

**
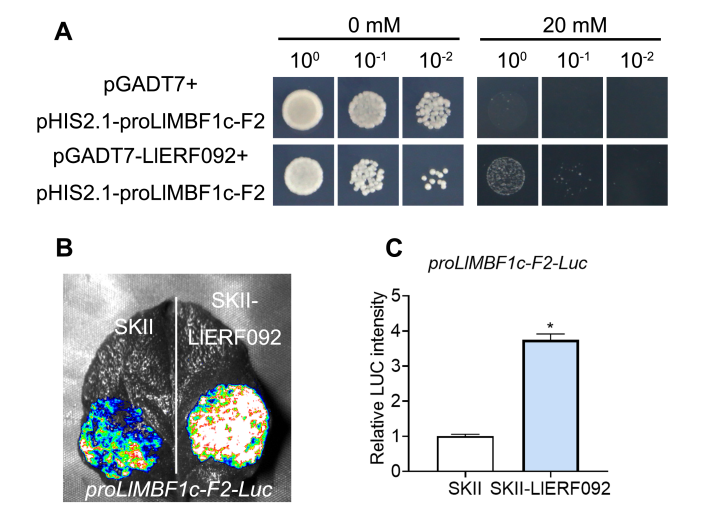
**

**Fig. S2.** LlERF092 binds to the F2 fragment of the *LlMBF1c* promoter and activated its expression. (A) Growth status of transformed yeast strains on SD/-Trp-His-Leu medium with varying concentrations of 3-amino-1,2,4- triazole (3-AT). SD, synthetic dropout medium. (B) LlERF092 activated the activity of the F2 fragment from the *LlMBF1c* promoter in *N. benthamiana*. (C) Measurement of the relative fluorescence intensity in (B). Data are shown as the mean ± SD of three replicates (Student’s *t*-test, *P < 0.05).


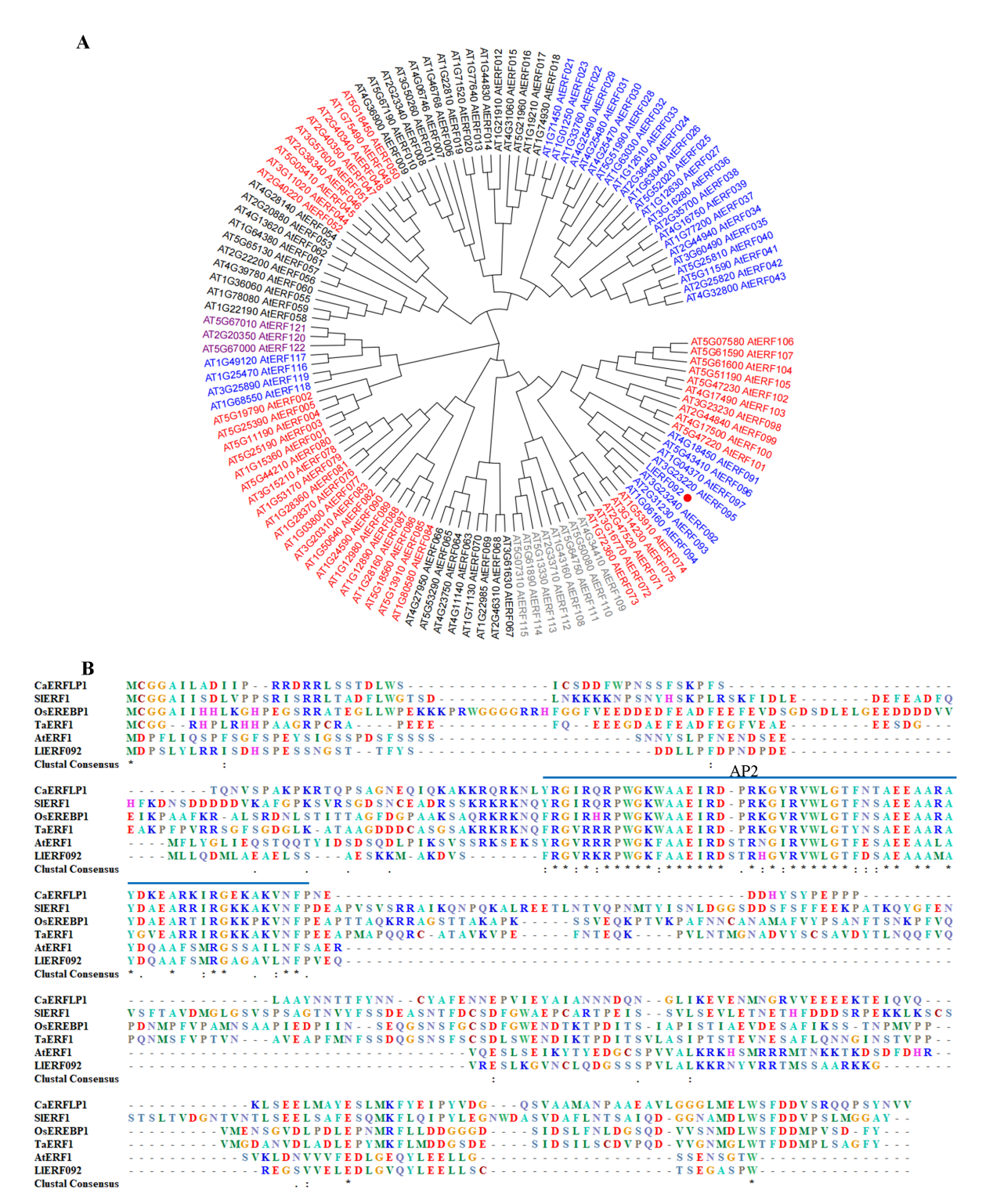


**Fig. S3.** Sequence analysis of LlERF092. (A) Phylogenetic analysis of LlERF092 and the members of the Arabidopsis ERF family. (B) Multiple comparisons of LlERF092 amino acid sequences. *Solanum lycopersicum* ERF1 (SlERF1); *Capsicum annuum* ERFLP1 (CaERFLP1); *Oryza sativa* EREBP1 (OsEREBP1); *Triticum aestivum* ERF1 (TaERF1); *Arabidopsis thaliana* ERF1 (AtERF1); *Lilium longiflorum* ERF092 (LlERF092).

**
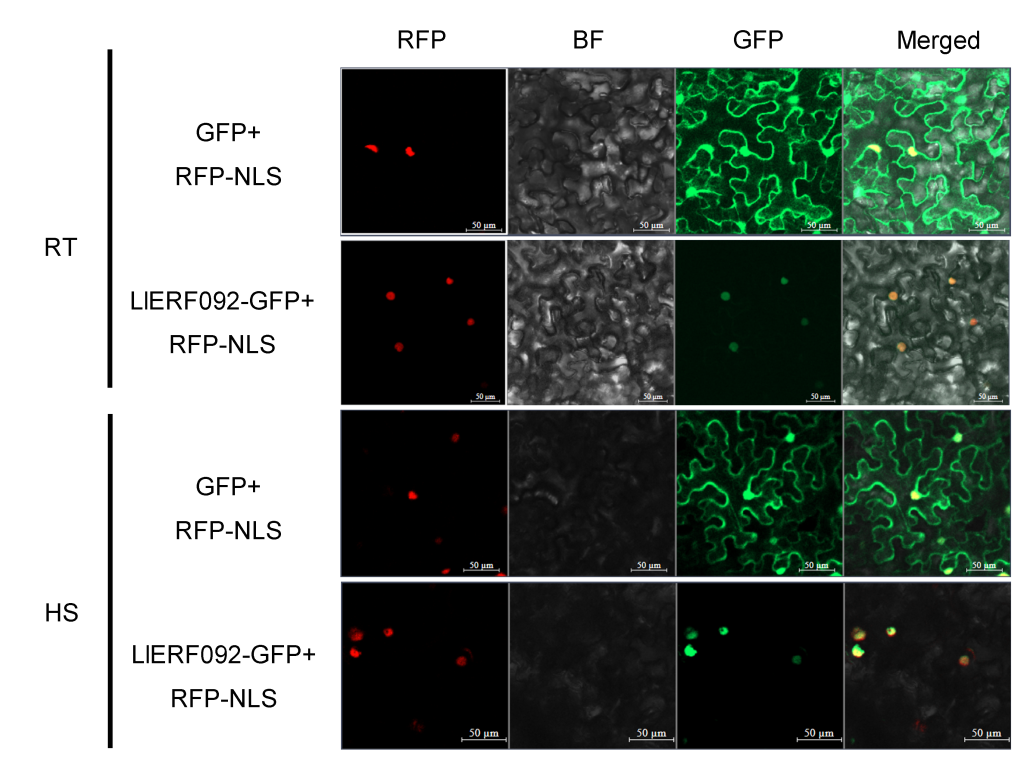
**

**Fig. S4.** Subcellular localization of LlERF092. RFP, the red fluorescence protein; BF, the bright light; GFP, green fluorescence protein; Merged, the overlay plots; RT, 22℃; HS, 37℃ for 30 min. Scale Bars = 50 μm.


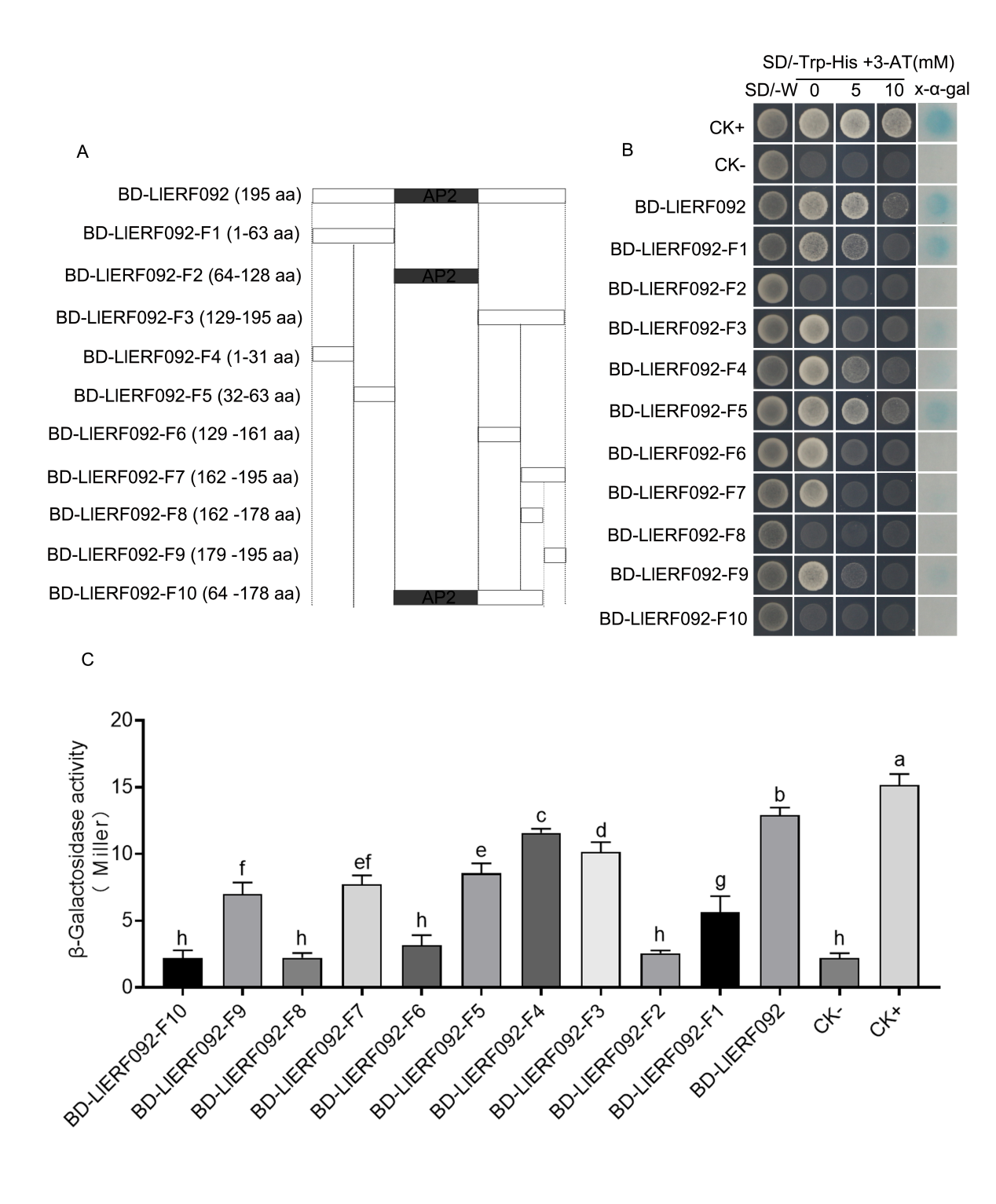


**Fig. S5.** Transactivation activity assay in the yeast cells. (A) Schematic diagram of vector construction. (B) Analysis of the growth status of yeast cells. (C) Analysis of β-galactosidase activity. GAL4 served as the positive control (CK+); BD was used as the negative control (CK-). Data are shown as the mean ± SD of three replicates, with different letters indicating statistically significant difference (Student-Newman-Keuls test, P < 0.05).


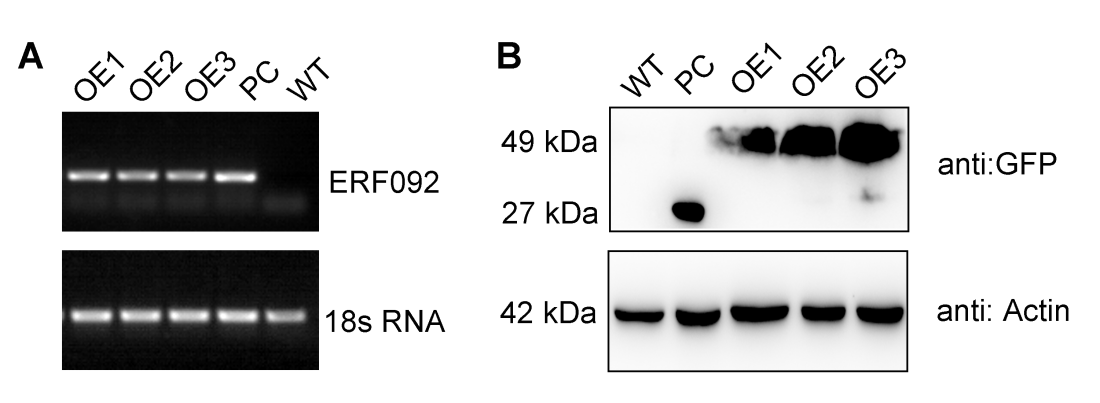


**Fig. S6.** Identification of *LlERF092* overexpression lines. (A) Detection of *LlERF092* overexpression lines at DNA levels. PC: positive control, the LlERF092-GFP plasmid served as positive control; WT, wild type. (B) Detection of *LlERF092* overexpression lines at protein levels. PC, positive control, GFP protein served as positive control; WT, wild type.


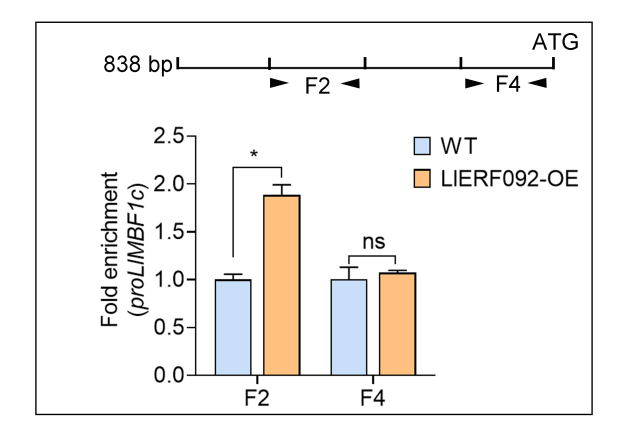


**Fig. S7.** LlERF092 binds to the F2 fragment of the *LlMBF1c* promoter in *LlERF092-*OE2 transgenic line. Data are presented as mean ± SD of three replicates (Student’s *t*-test, *P < 0.05); ns, no significant difference.


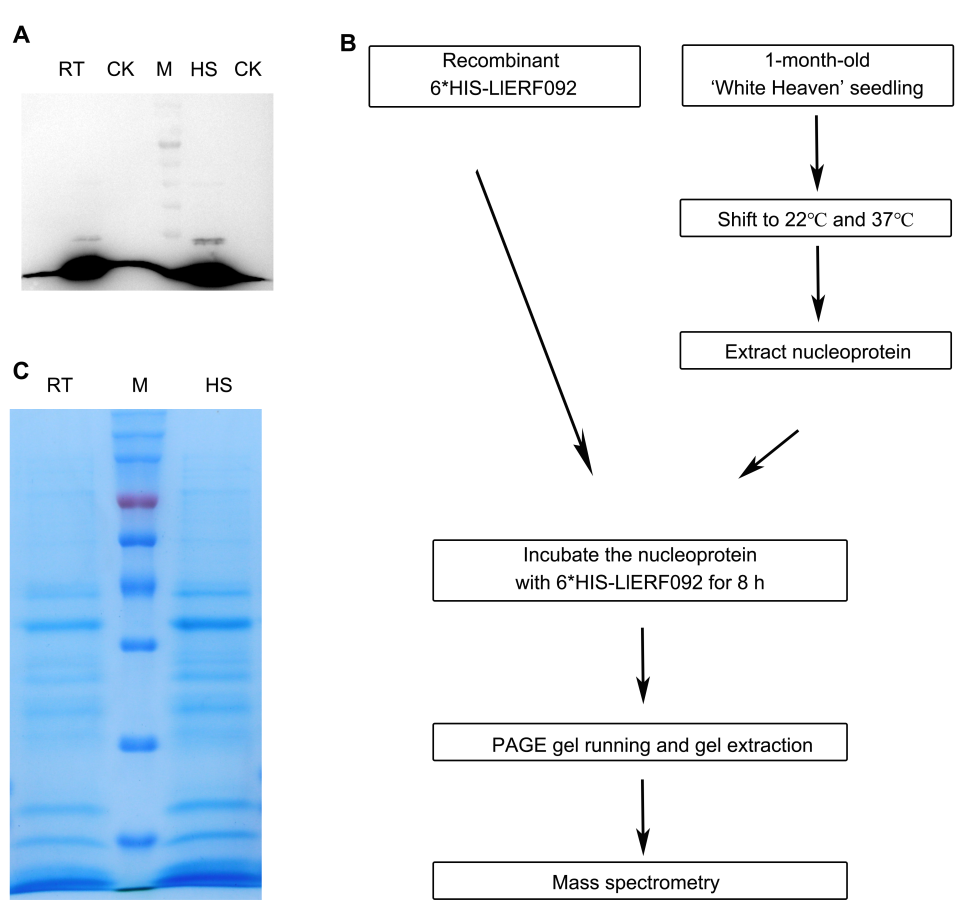


**Fig. S8.** IP-MS screening of LlERF092 interaction proteins. (A) Immunoblotting assay of Histone 3 of nuclear protein extraction from ‘White Heaven’. (B) Schematic diagram of screening the LlERF092 interaction proteins by immunoprecipitation-mass spectrometry technique. (C) SDS-PAGE gel electrophoresis of HIS pull-down assay of LlERF092 with nuclear proteins.

**
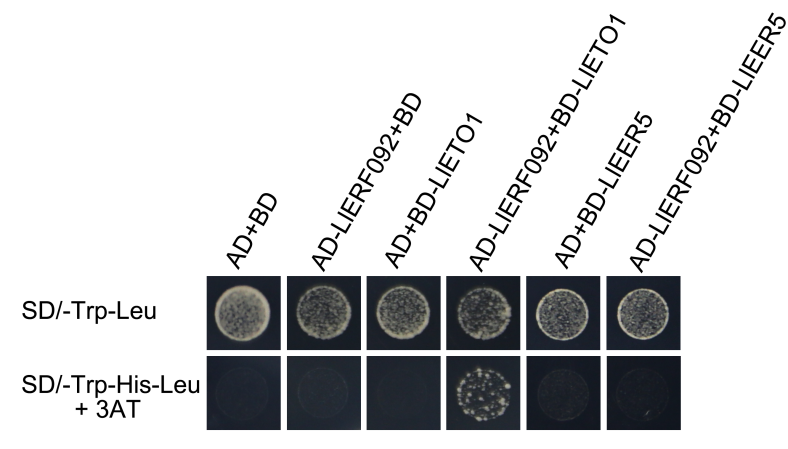
**

**Fig. S9.** Yeast two-hybrid assay of LlERF092 with LlETO1 and LlEER5. AD, pGADT7; BD, pGBKT7; SD, synthetic dropout medium. The representative picture came from three independent experiments.


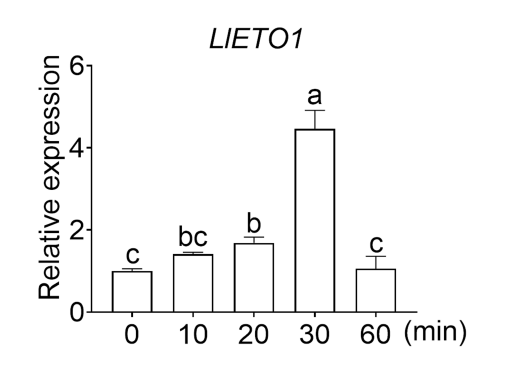


**Fig. S10.** Expression analysis of *LlETO1* under heat stress. The uniformly sized seedlings were treated with 37℃; 18s rRNA of lily served as a reference gene. Data are shown as the mean ± SD of three replicates, with different letters indicating statistically significant difference (Student-Newman-Keuls test, P < 0.05).


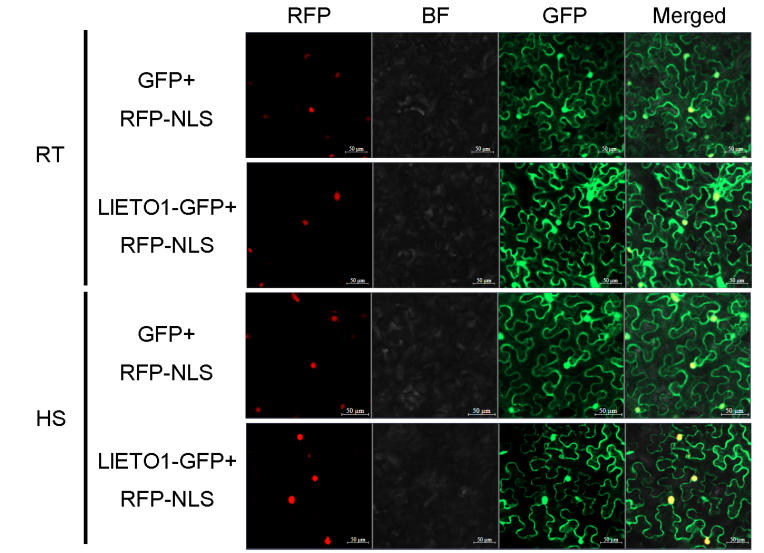


**Fig. S11.** Subcellular localization of LlETO1. RFP, red fluorescence protein; BF, the bright light; GFP, green fluorescence protein; Merged, the overlay plots. RT, 22℃; HS, 37℃ for 30 min. Scale Bars = 50 μm.


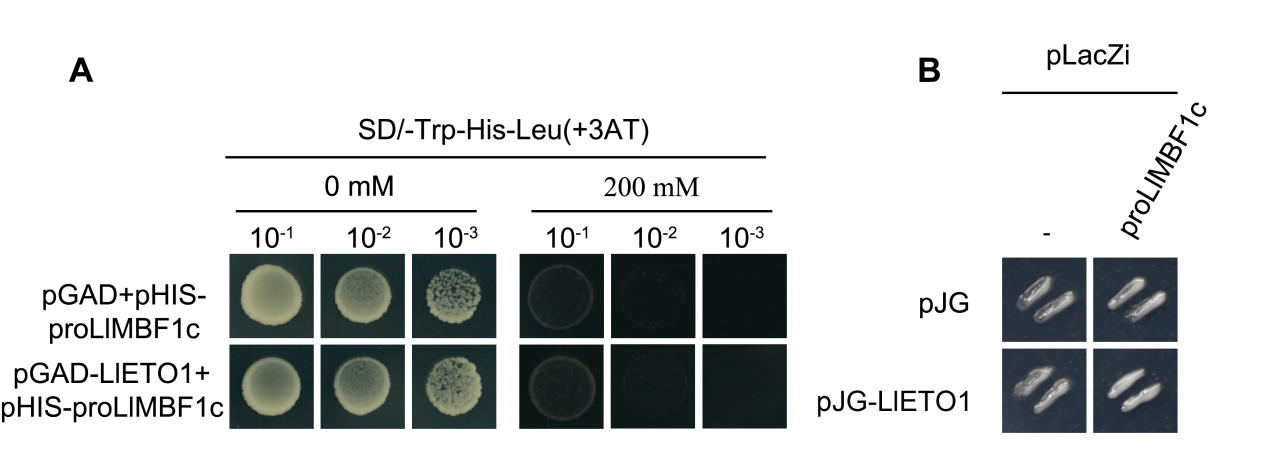


**Fig. S12.** Yeast one-hybrid analysis relates to the binding of LlETO1 to the *LlMBF1c* promoter. (A) Growth status of transformed yeast cells on SD/-Trp-His-Leu medium with different concentrations of 3-amino-1,2,4- triazole (3-AT). (B) Yeast one-hybrid analysis of the interaction between LlETO1 and the *LlMBF1c* promoter. Yeast cell growth on SD/-Ura-Trp deficient medium containing X-gal (5-bromo-4-chloro-3-indolyl β-d-galactopyranoside) was used to assess this interaction. SD, synthetic dropout medium.


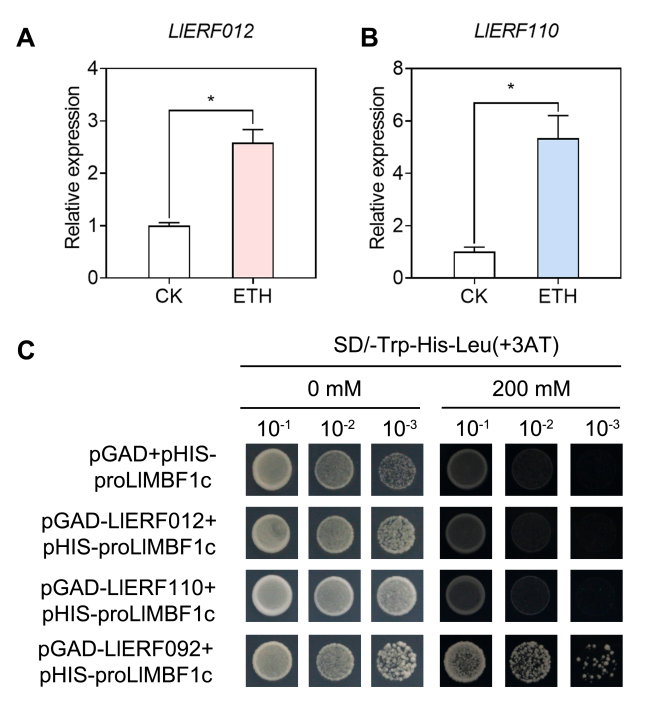


**Fig. S13** Expression analysis of *LlERF012* and *LlERF110* under ethephon treatment and yeast one-hybrid assay of LlERF012 and LlERF110 interaction with the *LlMBF1c* promoter. (A) Expression analysis of *LlERF012* under ethephon treatment. 18s rRNA of lily served as the reference gene. Data are the mean ± SD of three replicates (Student’s *t*-test, *P < 0.05). (B) Expression analysis of *LlERF110* under ethephon treatment. 18s rRNA of lily served as the reference gene. Data are the mean ± SD of three replicates (Student’s *t*-test, *P < 0.05). (C) Growth status of transformed yeast cells on SD/-Trp-His-Leu medium with different concentrations of 3-amino-1,2,4-triazole (3-AT). SD, synthetic dropout medium.
